# Effect of a comprehensive package based on combined C-reactive protein and serum amyloid A point-of-care testing on antibiotic prescribing for acute respiratory tract infections at village clinics in China: a cluster randomised controlled trial

**DOI:** 10.1016/j.lanwpc.2026.101888

**Published:** 2026-05-25

**Authors:** Minzhi Xu, Jing Wang, Zhitong Zhang, Erjia Ge, Yuting Zhu, Jinxi Li, Yanhong Gong, Zuxun Lu, Xiaolin Wei, Xiaoxv Yin

**Affiliations:** a Department of Social Medicine and Health Management, School of Public Health, Tongji Medical College, Huazhong University of Science and Technology, Wuhan, Hubei, China; b School of International Pharmaceutical Business, China Pharmaceutical University, Nanjing, Jiangsu, China; c Dalla Lana School of Public Health, University of Toronto, Toronto, ON, Canada

**Keywords:** Point-of-care testing, C-Reactive protein, Serum amyloid A, Antibiotic prescribing, Acute respiratory tract infections, Village clinics

## Abstract

**Background:**

Antibiotic misuse is a key driver of antimicrobial resistance globally, with high rates of inappropriate use observed in rural China. We evaluated the effectiveness of a comprehensive package based on quantified point-of-care testing (POCT) for C-reactive protein and serum amyloid A (CRP&SAA) in reducing inappropriate antibiotic use for acute respiratory infections (ARIs) in village clinics in rural China.

**Methods:**

We conducted a pragmatic, parallel-group, cluster-randomised controlled trial at 40 village clinics in Xiantao City, Hubei Province, China, over a 6-month intervention period. Village clinics were randomly assigned (1:1) to provide a comprehensive package based on CRP&SAA POCT, or usual care. Randomisation was stratified by township. Eligible consultations were patients of all ages with a diagnosis of ARI or acute respiratory symptoms in village clinics. The primary outcome was the proportion of initial patient consultations diagnosed with ARIs resulting in any antibiotic prescriptions from village doctors in the intention-to-treat population. The 30-day hospital admission rate for ARIs or sepsis following the initial ARI consultation was measured as a safety outcome. This trial is registered with ClinicalTrials.gov, NCT06568432.

**Findings:**

Forty village clinics were randomly assigned, 20 to the intervention group (n = 17,754) and 20 to the control group (n = 17,354). A total of 7.4% consultations (1311 out of 17,754) performed the test in the intervention group. A total of 9001 (50.70%) consultations in the intervention group and 11,715 (67.51%) consultations in the control group resulted in antibiotic prescriptions (adjusted relative risk 0.80 [95% CI 0.78–0.82; p < 0.001]; adjusted risk difference −13% [95% CI −15 to −12; p < 0.001]). The 30-day hospitalisation rate for ARIs or sepsis did not differ significantly (0.32% in the control group versus 0.35% in the intervention group; adjusted relative risk 0.94 [95% CI 0.59–1.48; p = 0.78]; adjusted risk difference 0% [95% CI 0–0; p = 0.78]).

**Interpretation:**

The interventions can safely and effectively reduce antibiotic use among patients with ARIs in village clinics in rural China. Future implementation efforts should further examine the barriers and facilitators to adopting POCT within specific health system contexts and service delivery settings, and identify cost-effective strategies to scale up POCT in primary care.

**Funding:**

The University of Toronto-Huazhong University of Science and Technology JOINT SEED FUND and the Medical Young Top-notch Talent Cultivation Program of Hubei Province.

**Research in context**

**Evidence before this study**

A 2022 Cochrane review of 13 randomised controlled trials—12 trials (10,218 participants in total, of whom 2335 were children) evaluated a C-reactive protein point-of-care test (CRP POCT), and one trial (317 adult participants) evaluated a procalcitonin POCT—conducted in Europe, Russia, and Asia, and published between 1995 and 2021, showed that the use of CRP POCT as an adjunct to standard care likely reduces the number of primary care patients presenting with symptoms of acute respiratory infection (ARI) who are prescribed antibiotics. We searched PubMed, Cochrane, Embase, and Google Scholar using a combination of the terms “antibiotics”, “antibiotic prescribing”, “primary care”, “respiratory infections”, “respiratory diseases”, “point-of-care test”, “POCT”, “RCT”, and “randomised controlled trial” for articles published in English from 1 January 1990 to 28 February 2026. We identified ten additional trials conducted after the publication of the systematic review, spanning Vietnam, Uganda, Nigeria, Kyrgyzstan, Australia, France, Latvia, Spain, and Belgium. Among the published randomised controlled trials, four employed a pragmatic design in low- and middle-income countries: Vietnam, Uganda, Nigeria, and Kyrgyzstan. These trials have demonstrated the effectiveness of CRP POCT in primary healthcare settings. There is no randomised controlled trial evaluating the effectiveness of combined use of CRP and serum amyloid A (SAA) POCT on antibiotic prescribing for ARIs.

**Added value of this study**

This pragmatic, cluster-randomised trial evaluated the effect of a comprehensive package based on CRP&SAA POCT on antibiotic prescribing for ARIs in primary care facilities in rural China. Our intervention reduced antibiotic prescribing for ARIs by 13 percentage points. We also observed significant reductions in multiple antibiotic use and intravenous antibiotic administration. Moreover, we conducted the study under real-world implementation conditions—where the POCT has been incorporated into the routine health insurance scheme with a co-payment arrangement. Our trial provided a reliable, real-world evaluation of a comprehensive package based on CRP&SAA POCT on antibiotic use for ARIs in a resource-limited setting. The findings provide policy implications to low- and middle-income countries experiencing high antibiotic use, offering valuable insights for optimising antibiotic management.

**Implications of all the available evidence**

The available evidence, including this trial, shows the potential of biomarker POCT to safely and effectively reduce antibiotic use among patients with ARIs. Future implementation efforts should further examine the barriers and facilitators to adopting POCT within specific health system contexts and service delivery settings, and identify cost-effective strategies for scaling up POCT in primary care.

## Introduction

In rural China, antibiotic prescribing remains highly prevalent.^1^ Evidence indicates that antibiotics are prescribed in over half of outpatient consultations at village clinics, a rate substantially higher than that in higher-level healthcare facilities.^2^^,^^3^ Acute respiratory infections (ARIs) are the leading cause of antibiotic use in these clinics.^4^ The majority of ARIs are viral in origin, making antibiotics ineffective for most patients.^5^^,^^6^ Unnecessary antibiotic prescriptions not only increase the risk of adverse events and healthcare costs but also contribute to the escalating use of antibiotics.

Distinguishing bacterial from viral ARIs remains clinically challenging due to significant symptom overlap, which complicates diagnostic decision-making and is a major driver of inappropriate antibiotic prescribing.^7^ With the rapid development of point-of-care testing (POCT) technologies, growing evidence supports biomarker-guided POCT, such as C-reactive protein (CRP), as a strategy to improve diagnostic accuracy and promote rational antibiotic prescribing in ARIs.^8^^,^^9^ Nevertheless, several clinical trials have shown that CRP POCT, when used as a standalone intervention, has a limited impact on reducing antibiotic prescription rates for ARIs in primary care.^6^^,^^10^ While CRP POCT aids in infection assessment, its specificity is limited by elevation in viral infections. Moreover, because CRP reflects a dynamic inflammatory response, a single measurement may not reliably distinguish bacterial from viral infections, increasing the risk of delayed antibiotic initiation.^11^ A Cochrane review called for additional studies on novel biomarkers to inform antibiotic prescribing in primary care.^12^ Serum amyloid A (SAA) is an acute-phase protein that exhibits a rapid and pronounced increase specifically in response to viral infections, often within hours of symptom onset.^13^^,^^14^ This kinetic and magnitude profile contrasts with that of CRP, which typically rises more slowly and demonstrates less discrimination between viral and bacterial triggers in the early stages of ARIs. When combined with CRP, SAA improves the differentiation between viral and bacterial infections, addressing a key limitation of single-marker strategies that lack specificity for viral infections.^15^ This dual-marker approach may help to reduce diagnostic uncertainty in primary care settings where clinical signs alone are insufficient. Given the high rate of antibiotic prescribing for likely viral ARIs in rural China, a strategy that supports more confident early discrimination of viral cases through clear and interpretable biomarker guidance could provide meaningful benefits for antimicrobial stewardship. However, evidence on how to integrate biomarker POCT into real-world rural primary care systems remains scarce, limiting translation into national guidelines. This gap is particularly pertinent in the context of Chinese village clinics, where POCT has not previously been available, and village doctors often lack formal training in interpreting biomarker results or applying them to clinical decision-making. Simply providing the CRP&SAA POCT device without additional support, such as training and education, is unlikely to ensure its appropriate use or to reduce unnecessary antibiotic prescriptions. Therefore, the effective integration of CRP&SAA POCT in this setting requires a comprehensive package of practical supports tailored to local capacity and routine workflows.

To support policymakers in considering guideline updates and the large-scale implementation of CRP&SAA POCT in village clinics, we developed a comprehensive intervention package. This package, centred on CRP&SAA POCT, includes targeted training for village doctors on biomarker-guided management of ARIs, desk guide reminders, monthly performance feedback on adherence to POCT-based prescribing thresholds, a patient payment model, and patient education materials. All components are specifically designed to promote the appropriate use of the tests and aim to reduce inappropriate antibiotic use for ARIs in village clinics. We then tested its effectiveness in a cluster-randomised controlled trial, which we report here.

## Methods

### Study design and setting

We conducted a pragmatic, parallel-group, cluster-randomised controlled trial in 40 village clinics across 15 townships and 5 communities in Xiantao, Hubei Province, central China. The study area covered a residential population of 1.11 million. This trial compared a comprehensive package based on CRP&SAA POCT with usual care to guide antibiotic prescribing for ARIs. Data were collected over a six-month period. Village clinics serve as the primary care providers in rural China, offering essential services such as basic acute care, immunisation, maternal and child care, and chronic disease management to rural populations within a defined catchment area. Each village clinic is staffed by a village doctor and one nurse. Village doctors in rural China received three to five years of vocational medical training at secondary or tertiary technical colleges. Unlike physicians who graduated from a five-year medical education from a medical college, village doctors can not practice outside of their village, and often have limited knowledge of evidence-based guidelines for the management of ARIs. This context distinguishes them from primary care physicians in higher-income settings and emphasises the need for structured, practical stewardship support tailored to their training level and practice environment.

Prior to initiating the main trial, we conducted an internal pilot process to evaluate the feasibility of the overall study procedures. Eight village clinics across four townships were enrolled as pilot sites and randomised to either the intervention (a comprehensive package based on CRP&SAA POCT) or control group (usual care). All sites were followed up for one month. After confirming that the trial processes were feasible and acceptable, meeting our targets that over 75% of participating village doctors in the intervention group independently performed CRP&SAA POCT correctly and applied the stratified test results accurately to guide antibiotic use in more than 70% of consultations involving these tests. Then we initiated the main trial. No changes were made to study implementation processes between the pilot and main stages.

The trial has obtained ethical approval from the ethics committee of Huazhong University of Science and Technology (Ref: 2024S129). A waiver of written informed consent from patients to participate in the study was obtained from the relevant ethical review boards to minimise disruption to routine practice. Oral informed consent was obtained from all participants in accordance with the protocol approved by the ethics committee. The trial protocol has been previously published.^16^

### Eligibility

Inclusion criteria for village clinics included an annual outpatient volume exceeding 2000, a minimum of 10 ARI patients per week, and the presence of licenced prescribers. These criteria were validated using historical prescription records from the preceding year, obtained from the Xiantao City Health Commission's Electronic Medical Records.

The target population comprised (1) patients of all ages diagnosed by a village doctor with ARIs, including both upper and lower respiratory tract infections; and (2) patients presenting with one or more acute respiratory symptoms, such as cough, rhinitis (sneezing, nasal congestion, or a runny nose), sore throat, shortness of breath, wheezing, or abnormal auscultation findings. Patients with non-respiratory diseases, or those presenting with severe clinical features and assessed by village doctors as requiring referral to a higher-level facility (such as acute exacerbations of chronic obstructive pulmonary disease or pulmonary heart disease), were excluded. Both upper and lower respiratory tract infections were included to reflect actual clinical practice in village clinics, where antibiotics are commonly prescribed across the full spectrum of ARIs, including mild and likely viral cases. Although the majority of ARIs managed at village clinics are upper respiratory tract infections, CRP&SAA POCT remains a relevant tool to support appropriate antibiotic prescribing in this high-prescribing context. Our approach aligns with a previous trial conducted in comparable settings, which adopted the broad inclusion of all ARIs was adopted to address systematically high baseline antibiotic usage.^8^

The Xiantao City Health Commission facilitated data linkage by connecting the village clinic electronic health record with all hospitals in Xiantao City, enabling the identification of consequent hospitalisations. To protect patient confidentiality, the City Health Commission applied a secure algorithm to generate a unique study identifier for each patient.

### Randomisation and masking

The 40 village clinics were randomly assigned (1:1) to either the intervention or control group using stratified permuted block randomisation, stratified by township. Randomisation was conducted using a computer programme written in R (version 4.0.5). Due to the nature of the intervention, blinding of both patients and village doctors was not feasible. To minimise outcome assessment bias, the trial adopted the “PROBE” design, in which data analysts were unaware of group allocation.^17^

### Intervention procedures

Following randomisation, the Serum Amyloid A and C-Reactive Protein Combined Assay Kit (a quantitative immunochromatographic test) was supplied to all village clinics in the intervention group. The Combined Assay Kit requires a finger-prick capillary blood sample of approximately 10–15 μL. The test is performed at the point of care by village doctors using a portable reader, with results available within 5 min. According to the manufacturer's specifications, the assay demonstrates strong linearity (r ≥ 0.997 over clinically relevant ranges) for both CRP and SAA when compared with standard laboratory methods. The procedure does not require refrigeration of reagents or specialised laboratory infrastructure, making it suitable for use in resource-limited primary care settings. CRP&SAA POCT was recommended for all patients with ARIs. Antibiotic prescribing decisions were guided by a pre-specified algorithm based on combined CRP and SAA levels: antibiotics were recommended only when SAA >100 mg/L with CRP ≥10 mg/L, or when CRP >50 mg/L regardless of SAA; in all other cases, antibiotics were not recommended (Appendix, pp. 7). Final decisions remained at the discretion of the village doctor, based on clinical assessment. Current international guidelines recommend adjusting biomarker interpretation in the presence of certain comorbidities. However, such conditions are not regularly documented or accessible to village doctors in rural China. Our trial adopted a pragmatic design to evaluate the intervention under routine practice conditions, where village doctors do not routinely assess or adjust for chronic comorbidities when managing ARIs. Consequently, the decision algorithm was simplified to enhance feasibility and generalisability in low-resource primary care settings.

To support effective implementation, the intervention package was guided by the Theoretical Domains Framework (TDF), an integrative framework derived from behavioural change theories.^18^^,^^19^ We selected key theoretical domains within the TDF that were considered relevant to the implementation of CRP&SAA POCT, and mapped these to specific behaviour change techniques to inform the design of the intervention (Appendix, pp. 2–3). All village doctors in the intervention group received face-to-face training on the use of CRP&SAA POCT, supplemented by clinical guidelines and desk guide reminders. Specifically, centralised, in-person training was provided to ensure all village doctors in the 20 intervention clinics were fully aware of the test availability and its role in the management of ARIs. The training covered: (1) the operational procedures for performing CRP&SAA POCT; (2) recommended thresholds for CRP&SAA, along with practical case applications; and (3) clinical evaluation and management of ARIs. Furthermore, we have developed clinical guidelines based on the latest Chinese and international standards for antibiotic use.^20^^,^^21^ These guidelines primarily target ARIs. The recommended thresholds for CRP and SAA in our guidelines were adapted from a synthesis of previous global studies^6^^,^^8^ and expert consensus in China^22^ to better align with local clinical practice and the specific test kit used. The clinical guidelines were presented in the form of a manual, with the main contents including: (1) an introduction to ARIs; (2) standardised procedures for the diagnosis and treatment of ARIs; (3) clinical diagnosis and treatment pathways for ARIs based on CRP&SAA POCT; and (4) a brief overview of the principles of CRP&SAA POCT, including illustrated operational steps: doctors are required to offer the test to eligible ARI patients, perform the finger-prick assay using the portable reader, and await the results before finalising prescribing decisions. The desk guide reminders were provided on sticky notes displaying the recommended thresholds for CRP and SAA levels (mg/L), helping village doctors more easily guide antibiotic use based on these thresholds. Each month, prescription data were reviewed from intervention clinics to assess adherence to the predefined CRP&SAA POCT thresholds. Feedback on performance was subsequently provided to each clinic and administrative personnel. Patient education materials were distributed in the form of a popular science leaflet to enhance patients’ understanding of how CRP&SAA POCT support diagnostic and treatment decisions for ARIs. The leaflet also emphasised the role of biomarker-guided prescribing in promoting the rational use of antibiotics and the potential harms associated with antibiotic overuse. To simulate real-world implementation conditions and incentivise village doctors to use CRP&SAA POCT, a patient-payment model was implemented. We incorporated the cost of the test into the health insurance scheme, where patients paid 5 RMB (≈0.71US$) per test, covering 40% of the total price, while the insurance covered the remaining amount. The 5 RMB (≈0.71US$) was given to village doctors as an incentive according to the trial design.

### Control procedures

Village clinics assigned to the control group continued to diagnose and manage ARIs according to usual care practices. Antibiotic prescribing was determined by village doctors based on their existing knowledge and clinical judgement, without any additional training on antibiotic use. No health education materials were provided to patients with ARIs, and the village clinics did not receive access to the Serum Amyloid A and C-Reactive Protein Combined Assay Kit.

### Outcomes

The primary outcome was the proportion of initial patient consultations diagnosed with ARIs resulting in any antibiotic prescriptions from village doctors. An initial patient consultation was defined as a visit to the current village clinic without any recorded attendance in the preceding two weeks. Since most ARIs are self-limiting and caused by viruses that do not require antibiotic treatment, a reduction in the prescribing rate reflects more rational antibiotic use.^23^

To examine whether the intervention influenced the patterns of antibiotic prescribing, we included two secondary outcomes. The secondary outcomes were the proportions of ARI patients receiving multiple antibiotics and receiving intravenous antibiotics during their initial consultation. Additionally, the proportion of ARI patients receiving any Traditional Chinese Medicine prescription during their initial consultation was included as a secondary outcome. This was based on findings from our previous trial, which showed an increase in Traditional Chinese Medicine use that may reflect its role as a potential alternative to antibiotics in the management of ARIs.^23^ To assess whether the intervention affected patient healthcare costs, we included two additional secondary outcomes: the mean cost of medications and the mean cost per consultation. Finally, to monitor potential undertreatment, we included the 30-day hospital admission rate in Xiantao City for ARIs or sepsis following the initial ARI consultation as a safety outcome.

### Statistical analysis

Based on our exploratory study and previous evidence, we estimated that the antibiotic prescribing rate for ARIs in rural primary care in China would be 50%.^3^^,^^24^

Based on our previous trial we assumed that the intra-cluster correlation coefficient would be 0.05.^23^ We hypothesised that the intervention would achieve a relative reduction of 25% in antibiotic prescribing rates. To detect this effect with 90% power at a two-sided 5% significance level, assuming an average cluster size of 500 patients and accounting for an estimated 15% loss of prescriptions, 20 village clinics per arm were required. Therefore, we included 40 village clinics in total.

All analyses adhered to the intention-to-treat (ITT) principle, including all initial patient consultations diagnosed with ARIs in both the intervention and control groups. Our pre-defined per-protocol analysis for the primary outcome included only initial patient consultations diagnosed with ARIs in which CRP&SAA POCT were performed. As this selects a non-random, post-randomisation subset, it violates exchangeability; thus, results represent exploratory associations rather than causal effects. No interim analysis was done. Between-group relative risks (RRs) were estimated using generalised estimating equations (GEEs) with a Poisson distribution and a log link function. We altered our protocol-planned approach to estimating risk differences (RDs) directly via GEEs with identity links, opting instead for the marginal standardisation method due to universal model convergence issues encountered with our original approach (Appendix, pp. 4–6). Specifically, we fitted logistic GEE models with a binomial distribution and a logit link function. RDs and their 95% confidence intervals (CIs) were then derived using average marginal contrasts (i.e., predicted marginal probabilities).^25^ For the cost outcomes, GEEs with a Gaussian distribution and an identity link function were used. An exchangeable correlation structure was assumed at the village clinic level to account for within-cluster correlation, and robust standard errors were used for parameter inference. All the models above adjusted for village doctors' characteristics (sex, age, and education level), ARI patients' characteristics (age, sex, and type of ARI), and stratum (township). Prespecified subgroup analyses for the primary outcome were conducted using the same analytical approach as described above. All data were obtained from the Xiantao City Health Commission's Electronic Medical Records. This database is used for billing and insurance reimbursement, so fields for patient demographics, diagnosis, prescribed medications (including costs), and doctor identifiers are mandatory and therefore complete for every recorded consultation. To assess the robustness of the observed effects, we conducted covariate-sensitivity analyses for all outcomes, excluding all covariates except the stratum.

We conducted several exploratory analyses to assess the robustness of our findings. These included: (1) cluster-level weighted linear regression (weighted by cluster size) to account for unequal cluster sizes; (2) GEE with delete-one-cluster Jackknife correction to address potential small-sample bias given the limited number of clusters; (3) per-protocol analyses of secondary cost outcomes; and (4) post-hoc 1:1 propensity score-matched analysis (stratified by ARI type and patient age) on the primary outcome to control for baseline confounding. As these analyses were not pre-specified in the published trial protocol, they should be interpreted as exploratory.

Statistical analyses were performed using R (version 4.0.5; R Foundation for Statistical Computing) with the *geepack* package for GEE models. Two-sided p < 0.05 was considered statistically significant for all analyses. This trial is registered with ClinicalTrials.gov, NCT06568432.

### Role of the funding source

The funders of the study had no role in the study design, data collection, data analysis, data interpretation, manuscript writing, or the decision to submit.

## Results

**Fig. 1:**
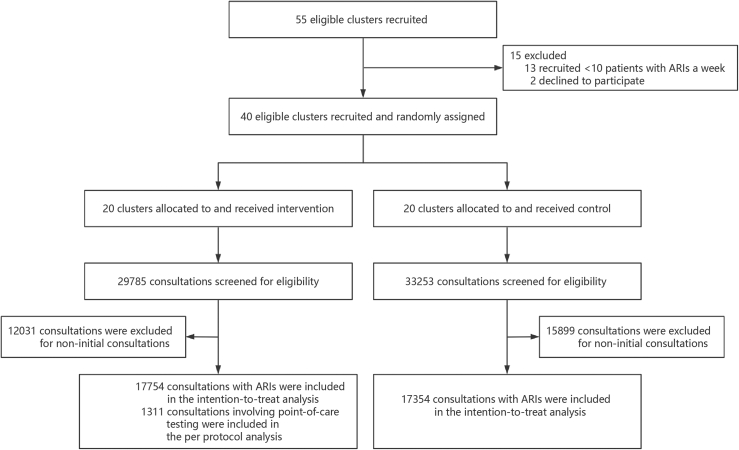
Trial profile.

We recruited 40 eligible village clinics in Xiantao City and randomly allocated 20 to the intervention group and 20 to the control group. We then implemented the intervention between February 15, 2025, and August 15, 2025. We included all eligible ARI consultations. At the end of the intervention period, the electronic health records identified a total of 63,038 eligible ARI consultations, of which 29,785 were in the intervention group and 33,253 were in the control group. After excluding the non-initial ARI consultations, we included a total of 35,108 initial ARI consultations for analysis. Among them, 17,754 were in the intervention group and 17,354 were in the control group (Fig. 1). Over the six-month study period, the number of initial ARI consultations per clinic ranged from 238 to 2784; The lowest-volume clinics in both groups had similar consultation counts (248 in the intervention group versus 238 in the control group), while the highest-volume clinic in the intervention group had slightly fewer consultations than its counterpart in the control group (2581 versus 2784) (Appendix, pp. 8). Overall, baseline characteristics of doctors and patients were well balanced between groups, with some modest (>5%) imbalances in patients' age and doctors’ education level (Table 1). We observed that the adoption rate of POCT was low, with only 7.4% (1311 out of 17,754) of initial ARI consultations in the intervention group undertaking the test. CRP&SAA POCT was used inconsistently across the clinics in the intervention group, with the number of tests per clinic ranging from 13 to 193 (Appendix, pp. 9).

**Table 1:** Baseline patient and doctor characteristics.

	Control group (n = 20 clusters)	Intervention group (n = 20 clusters)
Patients' characteristics		
Initial patient consultations diagnosed with ARIs	17,354	17,754
Age		
≤15	1983 (11%)	3220 (18%)
16–65	9704 (56%)	9765 (55%)
>65	5667 (33%)	4769 (27%)
Median (IQR)	58 (39–68)	55 (33–67)
Sex		
Male	8274 (48%)	8252 (47%)
Female	9080 (52%)	9502 (53%)
Type of acute respiratory infection		
Upper respiratory tract	16,802 (97%)	17,417 (98%)
Lower respiratory tract	552 (3%)	337 (2%)
Doctors' characteristics		
Age		
<60	14 (70%)	13 (65%)
≥60	6 (30%)	7 (35%)
Sex		
Male	17 (85%)	17 (85%)
Female	3 (15%)	3 (15%)
Education		
Bachelor's degree or associate degree	4 (20%)	6 (30%)
High school or vocational secondary school	16 (80%)	14 (70%)
Clinics' characteristics		
Initial consultations for ARIs		
>1000	4 (20%)	5 (25%)
≤1000	16 (80%)	15 (75%)

ARI = acute respiratory infection. Data are n, n (%), or median (IQR).

In the ITT analysis of the primary outcome, 9001 (50.70%) of 17,754 consultations in the intervention group and 11,715 (67.51%) of 17,354 consultations in the control group resulted in antibiotic prescriptions (adjusted RR 0.80 [95% CI 0.78–0.82]; adjusted RD −13% [95% CI −15 to −12]). In the exploratory per-protocol analysis, antibiotic prescribing in the 1311 intervention consultations that received CRP&SAA POCT was compared with that in the control group from the ITT analysis (adjusted RR 0.52 [95% CI 0.46–0.58]; adjusted RD −29% [95% CI −33 to −24]). In the ITT analysis of the secondary outcomes, we found that, compared with the control group, the rates of multiple antibiotic prescriptions (adjusted RR 0.40 [95% CI 0.35–0.46]; adjusted RD −6% [95% CI −7 to −5]) and intravenous antibiotic prescriptions (adjusted RR 0.69 [95% CI 0.67–0.72]; adjusted RD −13% [95% CI −15 to −12]) in the intervention group decreased significantly, while the rate of Traditional Chinese Medicine prescriptions increased significantly (adjusted RR 1.08 [95% CI 1.04–1.11]; adjusted RD 3% [95% CI 2–4]) (Table 2). In the ITT analysis of the costing outcomes, we found that, compared with the control group, the full consultation cost in the intervention group decreased significantly (adjusted mean difference: −0.04 [95% CI −0.08 to −0.01]). In comparison, the cost of medicines remained unchanged (adjusted mean difference −0.01 [95% CI −0.05–0.02]) (Table 3). In the exploratory per-protocol analysis of the costing outcomes, we found that the full consultation cost in the intervention group increased significantly compared with the control group (adjusted mean difference 0.79 [95% CI 0.67–0.91]), while the cost of medicines remained unchanged (adjusted mean difference −0.02 [95% CI −0.14–0.09]) (Table 3). The 30-day hospitalisation rate for ARIs or sepsis did not differ significantly (adjusted RR 0.94 [95% CI 0.59–1.48]; adjusted RD 0% [95% CI 0–0]). When we conducted covariate-sensitivity analyses, performed cluster-level analyses, applied GEE with a delete-one-cluster Jackknife correction, or performed a post-hoc 1:1 propensity score-matched analysis, our primary results remained robust (Appendix, pp. 10–16).

**Table 2:** Effectiveness of the comprehensive intervention package based on CRP&SAA POCT on outcomes.

Outcomes	Control group (n = 20 clusters)	Intervention group (n = 20 clusters)	Adjusted relative risk (95% CI)	p value	Adjusted risk difference (95% CI)	p value
Primary outcome^a^						
Antibiotic prescription rate						
Intention to treat	11,715/17,354 (67.51%)	9001/17,754 (50.70%)	0.80 (0.78–0.82)	<0.001	−13% (−15 to −12)	<0.001
Per protocol	11,715/17,354 (67.51%)	456/1311 (34.78%)	0.52 (0.46–0.58)	<0.001	−29% (−33 to −24)	<0.001
Secondary outcomes						
Multiple antibiotic prescription rate	2163/17,354 (12.46%)	584/17,754 (3.29%)	0.40 (0.35–0.46)	<0.001	−6% (−7 to −5)	<0.001
Intravenous antibiotic prescription rate	6874/17,354 (39.61%)	4785/17,754 (26.95%)	0.69 (0.67–0.72)	<0.001	−13% (−15 to −12)	<0.001
Traditional Chinese Medicine prescription rate	8301/17,354 (47.83%)	9013/17,754 (50.77%)	1.08 (1.04–1.11)	<0.001	3% (2–4)	<0.001
Patient safety indicator						
30-day hospitalisation for ARI or sepsis after initial visit	55/17,354 (0.32%)	63/17,754 (0.35%)	0.94 (0.59–1.48)	0.78	0% (0–0)	0.78

ARI = acute respiratory infection. Data are n/N (%). Adjusted relative risks (RR) and risk differences (RD) were estimated using generalised estimating equations. Models included fixed effects for treatment group, village doctor characteristics (sex, age, education level), patient characteristics (age, sex, type of acute respiratory infection), and township. Analyses were clustered by village clinic to account for within-clinic correlation.

a The intra-cluster correlation coefficient for the antibiotic prescription rate was 0.25.

**Table 3:** Effect of the comprehensive intervention package based on CRP&SAA POCT on prescription costs.

Cost	Control group (n = 20 clusters)	Intervention group (n = 20 clusters)	Adjusted mean difference (95% CI)	p value
Intention to treat				
Full consultation cost (US$)^a^	4.06 (1.97)	3.96 (2.14)	−0.04 (−0.08 to −0.01)	0.02
Medication cost (US$)	2.64 (1.85)	2.66 (2.14)	−0.01 (−0.05 to 0.02)	0.46
Per protocol				
Full consultation cost (US$)^a^	4.06 (1.97)	4.65 (1.79)	0.79 (0.67–0.91)	<0.001
Medication cost (US$)	2.64 (1.85)	2.58 (1.66)	−0.02 (−0.14 to 0.09)	0.71

Data are mean (SD). Adjusted intervention vs control mean difference results were estimated using generalised estimating equations. Models included fixed effects for treatment group, village doctor characteristics (sex, age, education level), patient characteristics (age, sex, type of acute respiratory infection), and township. Analyses were clustered by village clinic to account for within-clinic correlation.

a Full consultation cost includes medicines, treatment, tests and the consultation. US$ values are based on the currency exchange rate on December 17, 2025, in which 1US$ = 7.05RMB.

The difference in the primary outcome between the intervention and control groups was maintained in subgroup analyses (Table 4). Significant effect modification was observed by type of ARIs (p for interaction <0.001) and period (p for interaction <0.001).

**Table 4:** Effectiveness of the comprehensive intervention package based on CRP&SAA POCT on primary outcome by subgroup.

Subgroup	Control group (n = 20 clusters)	Intervention group (n = 20 clusters)	Adjusted relative risk (95% CI)	Adjusted risk difference (95% CI)	p_interaction_^b^
Patients' age group, years					0.29
≤15	1223/1983 (62%)	1503/3220 (47%)	0.75 (0.69–0.82)	−15% (−19 to −11)	
16–65	6535/9704 (67%)	4976/9765 (51%)	0.80 (0.78–0.82)	−14% (−16 to −12)	
>65	3957/5667 (70%)	2522/4769 (53%)	0.82 (0.79–0.85)	−12% (−15 to −10)	
Patient's sex					0.62
Male	5681/8274 (69%)	4234/8252 (51%)	0.81 (0.78–0.83)	−13% (−15 to −11)	
Female	6034/9080 (66%)	4767/9502 (50%)	0.80 (0.77–0.82)	−14% (−15 to −12)	
Type of acute respiratory infection					<0.001
Upper respiratory tract	11,372/16,802 (68%)	8782/17,417 (50%)	0.79 (0.78–0.81)	−14% (−15 to −13)	
Lower respiratory tract^a^	343/552 (62%)	219/337 (65%)	0.89 (0.77–1.01)	−12% (−23 to −2)	
Period					<0.001
15 February–15 May, 2025	6199/9443 (66%)	5832/9951 (59%)	0.95 (0.92–0.97)	−3% (−5 to −2)	
16 May–15 August, 2025	5516/7911 (70%)	3169/7803 (41%)	0.62 (0.60–0.64)	−26% (−28 to −24)	

Data are n/N (%). Adjusted relative risks (RR) and risk differences (RD) were estimated using generalised estimating equations. Models included fixed effects for treatment group, village doctor characteristics (sex, age, education level), patient characteristics (age, sex, type of acute respiratory infection), and township. Analyses were clustered by village clinic to account for within-clinic correlation. The subgroup variable was not included as a covariate.

a Township was not included as a covariate due to limited sample size, as adjustment could have led to invalid or unstable test results; The analysis included 14 clusters in the intervention group and 12 clusters in the control group.

b The P value for subgroup interaction was derived from the regression model fitted on the RR scale.

## Discussion

Our trial evaluated the effect of a comprehensive package based on CRP&SAA POCT on antibiotic prescribing for ARIs in primary care facilities in rural China. The results demonstrated that the intervention was effective. Compared with the control group, the intervention reduced antibiotic prescribing for ARIs by 13 percentage points (an absolute reduction) and by 20% (a relative reduction). We also observed significant decreases in multiple antibiotic use and intravenous antibiotic administration. Moreover, under real-world implementation conditions—where the POCT has been incorporated in the routine health insurance scheme under which there is a co-payment arrangement—the overall consultation costs in the intervention group decreased compared to the control group.

Our results regarding antibiotic prescription rates are consistent with previous trials from other countries on the use of biomarker POCT to improve antibiotic prescriptions for ARIs.^8^^,^^9^^,^^26^, ^27^, ^28^, ^29^, ^30^, ^31^ Among the published cluster randomised trials, three were conducted in low- and middle-income countries.^8^^,^^29^^,^^30^ Compared with the trial in Vietnam, we identified a larger reduction in antibiotic prescription rates.^8^ Unlike the intervention effect in the Vietnamese trial, which diminished over time, the intervention effect in our study gradually increased. This difference may be attributed to the fact that Chinese village doctors initially lacked experience with POCT and required a learning curve to become proficient.^32^ Notably, over 97% of our study population comprised upper respiratory tract infections. While prior evidence for CRP POCT has focused on lower respiratory tract infections,^9^^,^^26^ the significant reduction in antibiotic prescribing observed here suggests that adding SAA to CRP may enhance diagnostic precision in upper respiratory tract infections cases, where viral aetiologies dominate. Nonetheless, the effect may differ in lower respiratory tract infections, where bacterial infection risk is higher. Future research should explore the differential performance of CRP&SAA POCT across ARI subtypes to refine its application.

In many health contexts, doctors would need to find a substitute for antibiotics during the consultations. Similar to a previous study, we identified a significant increase in the use of Traditional Chinese Medicine for managing ARIs.^23^ In China, the widespread acceptance of Traditional Chinese Medicine as an alternative treatment for ARIs may have narrowed the gap between treatment expectations and actual prescribing, while preserving village doctors' income, thereby enhancing doctors’ adherence to interventions aimed at reducing antibiotic use. To examine this potential mechanism, our trial took Traditional Chinese Medicine and the cost of medicines as secondary outcomes. We observed that although antibiotic prescribing decreased and Traditional Chinese Medicine increased in the intervention group, there was no significant increase in medicine costs. Our findings highlight that Traditional Chinese Medicine may have served as a “buffer” in the successful implementation of antimicrobial stewardship interventions in China, helping to maintain continuity of care while mitigating resistance to behavioural change driven by concerns over income loss. Further research is needed to determine whether increased use of Traditional Chinese Medicine represents a rational substitution or a risk of new overuse. We also examined the impact of the intervention on broader prescribing patterns, specifically the rates of multiple antibiotic use and intravenous antibiotic administration. Both outcomes showed significant reductions in the intervention group. Notably, the decline in intravenous antibiotic use may help explain the observed reduction in total consultation costs, despite no significant change in medication costs alone. Since intravenous treatment incurs additional expenses, including consumables such as infusion devices and syringes, reducing the number of injections likely eliminates these extra costs, thereby lowering overall consultation costs. This finding implies that the POCT-based intervention may not only affect antibiotic prescribing patterns but also indirectly reduce healthcare costs by decreasing unnecessary injections, provided the test cost remains reasonable; however, this mechanism requires confirmation through formal cost-effectiveness analyses that explicitly account for diagnostic pricing and reimbursement policies. In terms of safety, our trial showed no significant difference in 30-day hospitalisation rates for ARIs or sepsis between the groups, consistent with previous trials.^8^^,^^33^

The study aligns with the current national priority of antibiotic control in China. Apart from its effectiveness, the key strength of the trial lies in its pragmatic design. We fully integrated the biomarker POCT into the daily diagnostic and treatment processes of the primary care system in rural China by embedding it within a comprehensive intervention package, rather than implementing it as a standalone test in an idealised or research-driven environment. Another strength of this trial is that it provided a reliable, real-world evaluation of the effect of a comprehensive package based on CRP&SAA POCT on antibiotic use for ARIs in a resource-limited setting. The implementation environment closely resembled that of many low- and middle-income countries with high rates of antibiotic use, making the research findings generalisable and providing valuable insights for optimising antibiotic management in similar health systems.

Our trial has several limitations. First, the lack of blinding may have led to behavioural reactivity, such as the Hawthorne effect.^34^ However, consultations data were extracted from the Xiantao City Health Commission's Electronic Medical Records, without additional on-site monitoring or manual data collection, thereby minimising disruption to routine care. Second, we did not collect data on patient-reported outcomes, such as time to symptom resolution, subsequent antibiotic use within 14 days, disease severity at day 7, or days absent from work or school. These measures are commonly used in efficacy-focused trials to assess potential harms associated with reduced antibiotic prescribing.^30^^,^^33^ Although an internal assessment of subsequent antibiotic use within 14 days, based on available electronic medical records, showed a statistically significant trend favouring the intervention, these data were not reported in detail due to incomplete capture of out-of-facility and over-the-counter antibiotic use, which could lead to an underestimation of total consumption. Given the pragmatic design of our study and the large scale of implementation across 40 village clinics, during which more than 35,000 eligible consultations were enrolled, systematic follow-up of individual patients was not feasible within routine primary care workflows. Instead, we defined our safety indicator as 30-day hospitalisation for ARIs or sepsis, reflecting severe clinical deterioration necessitating a higher level of care. Although this approach may not capture milder adverse consequences of withholding antibiotics, it provides reassurance regarding the safety of the intervention in real-world primary care settings. Third, we used 30-day hospitalisation for ARIs or sepsis within Xiantao City as a safety outcome. We did not include patients who might seek hospitalisation in the nearby big city of Wuhan. However, we believe that very few patients would travel to Wuhan for care, given the non-severe nature of the cases included. Fourth, although specific behaviour change techniques were implemented to promote the use of CRP&SAA POCT, uptake remained low. This limited adoption may have attenuated the intervention's overall effectiveness and diminished the robustness of the observed differences in costs. Our process evaluation identified several key barriers to implementing POCT. Village doctors consistently reported that the test added to an already heavy clinical workload by increasing consultation times, which was a particular challenge during peak respiratory seasons. We implemented the intervention in real-world setting where medical tests are covered by health insurance with co-payment from patients. In this setting, patients were required to pay a 5 RMB (≈0.71US$) co-payment, which, despite appearing modest, remained a financial barrier to some rural residents. The tests require patients to provide finger blood drops, which may further reduce their willingness to undergo testing. Furthermore, village doctors suggested that expanding the test panel to include additional indicators, such as a complete blood count, would enhance their confidence in diagnostic accuracy and increase their willingness to use the POCT. A detailed process evaluation aimed at better understanding the barriers and facilitators associated with the low uptake of CRP&SAA POCT will be reported separately.

There is an urgent global need for more effective technologies that are compatible with primary care systems, serving as valuable tools to reduce inappropriate antibiotic use for ARIs. This is particularly crucial in low- and middle-income countries, where antibiotic misuse is a significant problem. In rural China, village doctors face the dual pressures of diagnostic uncertainty and patient expectations, which frequently result in the excessive use of empirical antibiotics. This study demonstrates that although CRP&SAA POCT hold considerable potential to guide antibiotic use, their successful implementation requires adaptation to the local context. Given the heavy workload and limited consultation time in primary care settings—particularly during respiratory disease seasons—and the requirement for patients to bear the additional cost of POCT, scaling up POCT not only necessitates technological implementation but also faces significant challenges related to system integration. It is essential to incorporate POCT into the routine work of village doctors and to fully consider patients’ economic accessibility in order to enhance its acceptability and feasibility for broader implementation.

To conclude, our trial demonstrates that a comprehensive package based on quantitative POCT for CRP&SAA in village clinics in rural China can safely and effectively reduce antibiotic use among patients with ARIs. Future implementation efforts should further examine the barriers and facilitators to adopting POCT within specific health system contexts and service delivery settings, and identify cost-effective strategies for scaling up POCT in primary care.

## Contributors

XY, XW, MX, ZL, and YG contributed to the study design. XY, XW, JW, and MX developed the guideline and training. MX and JL prepared and cleaned the data. MX, JW, and YZ analysed the data. XY, XW, MX, YZ, JL, JW, YG, and ZL implemented the study. XY, XW, and MX took the lead in drafting the report. ZZ and EG provided substantial comments to improve the draft. JW verified the data and XY had access to raw data and XY had final responsibility for the decision to submit for publication. All authors contributed to the collection or interpretation of data, provided critical revisions to the report, and approved the final draft. XY and XW are the guarantors of the study.

## Data sharing statement

Individual patient data cannot be posted and downloaded in a public data depository and are not able to be transmitted out of China due to the National Privacy Regulation. Reasonable requests for data should be addressed to the corresponding author.

## Declaration of interests

We declare no competing interests.

## Appendix A Supplementary data

Supplementary Material

Protocol

## References

[1] Fu M., Gong Z., Zhu Y. (2023). Inappropriate antibiotic prescribing in primary healthcare facilities in China: a nationwide survey, 2017-2019. Clin Microbiol Infection.

[2] Lambert H., Shen X., Chai J. (2023). Prevalence, drivers and surveillance of antibiotic resistance and antibiotic use in rural China: interdisciplinary study. PLOS Glob Publ Health.

[3] Wang J., Wang P., Wang X., Zheng Y., Xiao Y. (2014). Use and prescription of antibiotics in primary health care settings in China. JAMA Intern Med.

[4] O'Connor R., O'Doherty J., O'Regan A., Dunne C. (2018). Antibiotic use for acute respiratory tract infections (ARTI) in primary care; what factors affect prescribing and why is it important? A narrative review. Ir J Med Sci.

[5] Pratt M.T.G., Abdalla T., Richmond P.C. (2022). Prevalence of respiratory viruses in community-acquired pneumonia in children: a systematic review and meta-analysis. Lancet Child Adolesc Health.

[6] Jung C., Levy C., Béchet S. (2024). Impact of C-reactive protein point-of-care testing on antibiotic prescriptions for children and adults with suspected respiratory tract infections in primary care: a French patient-level randomized controlled superiority trial. Clin Microbiol Infection.

[7] Laxminarayan R., Duse A., Wattal C. (2013). Antibiotic resistance—the need for global solutions. Lancet Infect Dis.

[8] Do N.T.T., Vu T.V.D., Greer R.C. (2023). Implementation of point-of-care testing of C-reactive protein concentrations to improve antibiotic targeting in respiratory illness in Vietnamese primary care: a pragmatic cluster-randomised controlled trial. Lancet Infect Dis.

[9] Boere T.M., van Buul L.W., Hopstaken R.M. (2021). Effect of C reactive protein point-of-care testing on antibiotic prescribing for lower respiratory tract infections in nursing home residents: cluster randomised controlled trial. BMJ.

[10] Schot M.J., Van den Bruel A., Broekhuizen B.D. (2018). Point-of-care C-reactive protein to assist in primary care management of children with suspected non-serious lower respiratory tract infection: a randomised controlled trial. BJGP Open.

[11] Levinson T., Wasserman A. (2022). C-Reactive protein velocity (CRPv) as a new biomarker for the early detection of acute infection/inflammation. Int J Mol Sci.

[12] Smedemark S.A., Aabenhus R., Llor C., Fournaise A., Olsen O., Jørgensen K.J. (2022). Biomarkers as point-of-care tests to guide prescription of antibiotics in people with acute respiratory infections in primary care. Cochrane Database Syst Rev.

[13] Perez L. (2019). Acute phase protein response to viral infection and vaccination. Arch Biochem Biophys.

[14] Malle E., DE Beer F.C. (1996). Human serum amyloid A (SAA) protein: a prominent acute-phase reactant for clinical practice. Eur J Clin Invest.

[15] Zou S., Liu J., Yang Z., Xiao D., Cao D. (2021). SAA and CRP are potential indicators in distinction and severity assessment for children with influenza. Int J Infect Dis.

[16] Xu M., Zhang Z., Ge E. (2025). Effect of C-reactive protein and serum amyloid A point-of-care testing on antibiotic prescribing for acute respiratory-tract infections at village clinics in China: a study protocol for a cluster randomised controlled trial. PLoS One.

[17] Hansson L., Hedner T., Dahlöf B. (1992). Prospective Randomized Open Blinded End-point (PROBE) Study. A novel design for intervention trials. Blood Press.

[18] Cane J., O'Connor D., Michie S. (2012). Validation of the theoretical domains framework for use in behaviour change and implementation research. Implement Sci.

[19] French S.D., Green S.E., O'Connor D.A. (2012). Developing theory-informed behaviour change interventions to implement evidence into practice: a systematic approach using the Theoretical Domains Framework. Implement Sci.

[20] NICE (2008).

[21] National Health and Family Planning Commission (2015). https://www.nhc.gov.cn/ewebeditor/uploadfile/2015/09/20150928170007470.pdf.

[22] Chinese Association of Integrated Traditional and Western Medicine: Laboratory Medicine Professional Committee (2019). Expert consensus on the clinical use of serum amyloid A in infectious diseases. Chin J Lab Med.

[23] Wei X., Zhang Z., Walley J.D. (2017). Effect of a training and educational intervention for physicians and caregivers on antibiotic prescribing for upper respiratory tract infections in children at primary care facilities in rural China: a cluster-randomised controlled trial. Lancet Glob Health.

[24] Zhang Z., Hu Y., Zou G. (2017). Antibiotic prescribing for upper respiratory infections among children in rural China: a cross-sectional study of outpatient prescriptions. Glob Health Action.

[25] Muller C.J., MacLehose R.F. (2014). Estimating predicted probabilities from logistic regression: different methods correspond to different target populations. Int J Epidemiol.

[26] Cals J.W.L., Butler C.C., Hopstaken R.M., Hood K., Dinant G.-J. (2009). Effect of point of care testing for C reactive protein and training in communication skills on antibiotic use in lower respiratory tract infections: cluster randomised trial. BMJ.

[27] Little P., Stuart B., Francis N. (2013). Effects of internet-based training on antibiotic prescribing rates for acute respiratory-tract infections: a multinational, cluster, randomised, factorial, controlled trial. Lancet.

[28] Llor C., Trapero-Bertran M., Sisó-Almirall A. (2024). Effects of C-reactive protein rapid testing and communication skills training on antibiotic prescribing for acute cough. A cluster factorial randomised controlled trial. NPJ Prim Care Respir Med.

[29] Onwunduba A., Ekwunife O., Onyilogwu E. (2023). Impact of point-of-care C-reactive protein testing intervention on non-prescription dispensing of antibiotics for respiratory tract infections in private community pharmacies in Nigeria: a cluster randomized controlled trial. Int J Infect Dis.

[30] Ciccone E.J., Hu D., Preisser J.S. (2024). Point-of-care C-reactive protein measurement by community health workers safely reduces antimicrobial use among children with respiratory illness in rural Uganda: a stepped wedge cluster randomized trial. PLoS Med.

[31] Isaeva E., Bloch J., Akylbekov A. (2025). C-reactive protein testing in primary care and antibiotic use in children with acute respiratory tract infections in Kyrgyzstan: an open-label, individually randomised, controlled trial. Lancet Reg Health Eur.

[32] Anand S., Fan V.Y., Zhang J. (2008). China's human resources for health: quantity, quality, and distribution. Lancet.

[33] Do N.T.T., Ta N.T.D., Tran N.T.H. (2016). Point-of-care C-reactive protein testing to reduce inappropriate use of antibiotics for non-severe acute respiratory infections in Vietnamese primary health care: a randomised controlled trial. Lancet Glob Health.

[34] McCambridge J., Witton J., Elbourne D.R. (2014). Systematic review of the hawthorne effect: new concepts are needed to study research participation effects. J Clin Epidemiol.

