# Supplementary material for: Effect of a comprehensive package based on combined C-reactive protein and serum amyloid A point-of-care testing on antibiotic prescribing for acute respiratory tract infections at village clinics in China: a cluster randomised controlled trial
Source: Lancet Reg Health West Pac. 2026 May 25;71:101888. doi: 10.1016/j.lanwpc.2026.101888 (PMC13227237; doi:10.1016/j.lanwpc.2026.101888)
Supplement: Supplementary Material [file mmc1.docx]

**Effect of a comprehensive package based on combined C-reactive protein and serum amyloid A point-of-care testing on antibiotic prescribing for acute respiratory tract infections at village clinics in China: a cluster randomised controlled trial**

**[Additional intervention process details](#_Toc17362)** [2](#_Toc17362)

**[Statistical analysis details](#_Toc11197)** [4](#_Toc11197)

**[Table S1.](#_Toc12575)** [CRP&SAA levels in those with an initial antibiotic prescription. 7](#_Toc12575)

**[Figure S1.](#_Toc16651)** [Number of initial consultations for ARIs per village clinic over a six-month period. 9](#_Toc16651)

**[Figure S2.](#_Toc10270)** [The frequency of POCT utilisation in the intervention group. 10](#_Toc10270)

**[Table S2.](#_Toc13009)** [Sensitivity analyses of the comprehensive intervention package based on CRP&SAA POCT effects on outcomes 11](#_Toc13009)

**[Table S3.](#_Toc23652)** [Sensitivity analyses of the comprehensive intervention package based on CRP&SAA POCT effects on prescription costs 12](#_Toc23652)

**[Table S4.](#_Toc12977)** [Cluster-level analysis of the effects of the comprehensive intervention package based on CRP&SAA POCT on outcomes 13](#_Toc12977)

**[Table S5.](#_Toc2975)** [Cluster-level analysis of the effects of the comprehensive intervention package based on CRP&SAA POCT on prescription costs 14](#_Toc2975)

**[Table S6.](#_Toc6797)** [Effectiveness of the comprehensive intervention package based on CRP&SAA POCT on outcomes: using generalised estimating equations with delete-one-cluster Jackknife correction to address potential small-sample bias 15](#_Toc6797)

**[Table S7.](#_Toc29456)** [Effect of the comprehensive intervention package based on CRP&SAA POCT on prescription costs: using generalised estimating equations with delete-one-cluster Jackknife correction to address potential small-sample bias 16](#_Toc29456)

# Additional intervention process details

To facilitate the implementation of CRP&SAA POCT, the intervention design is guided by the Theoretical Domain Framework (TDF), an emerging approach developed from a diverse array of theories related to behavior change. We presented a detailed table linking theoretical domains with interventions, and then proposed behaviour change in village doctors and patients in our protocol. The intervention design is summarised in the table below.

**Table** A comprehensive intervention package based on CRP&SAA POCT aimed at reducing antibiotic use in patients with ARIs in village clinics.

| **Target population** | **Theoretical domains** | **Behaviour change techniques, modes and content of delivery** |
| --- | --- | --- |
| Village doctors | Knowledge  Skills | Techniques: information provision  Mode 1: centralised in-person training.  Content: (1) the operational process for CRP&SAA POCT; (2) recommended thresholds for CRP&SAA, along with practical case applications; and (3) clinical evaluation and management of ARIs.  Mode 2: clinical guidelines manual.  Content: (1) an introduction to ARIs; (2) standardised procedures for the diagnosis and treatment of ARIs; (3) clinical diagnosis and treatment pathways for ARIs based on CRP&SAA POCT; (4) a brief overview of the principles of CRP&SAA POCT, along with illustrated operational steps.  Mode 3: desk guide reminders.  Content: the recommended thresholds from CRP&SAA |
|  |  |  |
|  | Reinforcement | Techniques: incentive.  Mode: charged for CRP&SAA POCT  Contents: each patient with an ARI utilising the CPR&SAA POCT will ultimately pay 5 RMB (approximately US $0.68) to the village clinic. |
|  | Behaviour regulation | Techniques: feedback and monitoring.  Mode: appraisal of antibiotic prescribing  Contents: (1) at the beginning of each month following the intervention, the research team will collect prescription information for ARIs from all village clinics within the intervention arm; (2) conduct a comprehensive assessment to determine whether village doctors are prescribing antibiotics in accordance with the CRP&SAA POCT threshold; (3) provide feedback to the relevant village clinics and administrative staff. |
| ARI patients | Knowledge  Beliefs about consequences | Techniques: information provision.  Mode: health education  Content: a popular science leaflet outlines the role of CRP&SAA POCT in promoting the rational use of antibiotics for ARIs, as well as the risks associated with antibiotic overuse. |
|  |  |  |

Abbreviation:CRP, C-reactive protein; SAA, Serum amyloid A; POCT, point-of-care testing; ARIs, acute respiratory infections.

# Statistical analysis details

September 1, 2025

Authors: Minzhi Xu, Xiaolin Wei, Xiaoxv Yin

**2.1 Statistical software**

Statistical analyses were performed using R software, version 4.0.5 (R Foundation for Statistical Computing). The *geepack* package was used to fit generalised estimating equations (GEEs).

**2.2 Analysis populations**

The target population of this study included (1) patients of all ages diagnosed by a village doctor with ARIs (including upper and lower respiratory infections); and (2) patients who present with ≥1 acute respiratory symptoms (including cough, rhinitis (sneezing, nasal congestion or runny nose), sore throat, shortness of breath, wheezing or abnormal auscultation).

**2.3 Baseline characteristics**

Baseline characteristics were summarised by randomised treatment arm as median (IQR) for continuous data and percentage for categorical data.

# 2.4 Primary outcome

The primary outcome is the proportion of patients who are diagnosed with ARIs and prescribed antibiotics during their initial visit (defined as no prescription record at the current institution within the preceding 14 days) in both study arms. This outcome serves as the primary indicator, reflecting the overall impact of a comprehensive intervention based on CRP&SAA POCT in guiding antibiotic use for patients with ARIs. Since most self-limiting ARIs are caused by viral infections that do not require antibiotic treatment, the decline in antibiotic prescribing rates suggests that village doctors are prescribing antibiotics more judiciously. The selection of this outcome is both feasible and reliable within the context of village clinics in China. This is due to the transition of prescriptions from traditional paper documents to electronic storage, which allows for the proper preservation of prescription records, thereby ensuring data integrity and traceability. The primary outcome was analysed in subgroups based on ARI patient’s age (1-15 / 16-65 / over 65 years old), ARI patient’s sex (male / female), type of ARI (upper respiratory tract infection / lower respiratory tract infection), and season.

**2.5 Secondary outcomes**

Secondary outcomes are extracted from the prescribing information related to the primary outcome, as well as from subsequent visit information. In particular, we included the proportion of participants using any form of Traditional Chinese Medicines as a secondary outcome. In our previous trials, we observed an increase in the use of Traditional Chinese Medicines, possibly as an alternative to antibiotics. These outcomes included the following types:

1. The proportion of multiple antibiotic prescriptions in the intervention and control arms (specifically, the proportion of ARI prescriptions that include two or more antibiotics).
2. The intravenously injected antibiotic prescription rate (the proportion that contain any antibiotics delivered by intravenous injection).
3. The proportion containing any Traditional Chinese Medicines.
4. The mean cost of an ARI prescription, based on the cost of any medicines.
5. The mean cost of a consultation, based on all costs including medicines, tests and the consultation.

**2.6 Patient safety indicator**

We evaluated whether the intervention increased the incidence of adverse events, which could occur if antibiotics were more frequently withheld for appropriate conditions as a result of the intervention. To address this concern, we used the proportion of patients with ARIs in both the intervention and control arms who were hospitalised in Xiantao City for ARIs or sepsis within 30 days after their initial ARI visit—excluding those referred by village doctors during the initial consultation—as a safety indicator.

**2.7 Missing data**

All data in this study were obtained from the Xiantao City Health Commission’s Electronic Medical Records and included patient-level information (age, sex, and diagnosis) as well as provider-level characteristics (village doctor’s age, sex, and educational level). As the data were extracted from an administrative database with complete records, there were no missing values in any of the variables analysed.

**2.8 Analysis methods**

To estimate relative risks (RRs), a Poisson GEE with a log link was applied. For continuous outcomes—such as the mean cost of an ARI prescription and consultation fee—linear regression with GEE was used, adjusting for within-clinic correlation. An exchangeable correlation structure was assumed at the village clinic level to account for within-cluster dependence, and robust standard errors were used for parameter inference.

We modified our protocol-specified approach for estimating crude and adjusted risk differences (RDs). Initially, we planned to use GEE with a binomial distribution and identity link, but encountered universal convergence issues—a well-recognised limitation of this method. As an alternative, we adopted a marginal standardisation approach. We fitted a logistic GEE model (binomial distribution with logit link) to account for clustering, and derived the adjusted RD and its 95% confidence interval using average marginal contrasts (i.e., predicted marginal probabilities). This approach provided stable estimation while maintaining interpretability on the absolute risk difference scale.

# 2.9 Codebook

| **VARIABLE NAME** | **MEANING** | **VALUE** |
| --- | --- | --- |
| intervention | group | 0=Usual care arm  1=CRP&SAA POCT arm |
| idd | village clinics | 40 village clinics (factor) |
| doc_age | The age of the village doctors | Continuous variable |
| doc_sex | The sex of the village doctors | 0=Female  1=Male |
| doc_edu_cata | The educational level of the village doctors | 0=High school or vocational secondary school  1=Bachelor’s degree or associate degree |
| pat_sex | The sex of the patients | 0=Female  1=Male |
| pat_age | The age of the patients | Continuous variable |
| aris_type | Type of acute respiratory infection | 0=Upper respiratory tract  1=Lower respiratory tract |
| doc_area | Townships | 20 townships (factor) |
| outcome_aiti | The proportion of patients who are diagnosed with ARIs and prescribed antibiotics during their initial visit | 0=No  1=Yes |
| outcome_multiple | The proportion of ARI prescriptions that include two or more antibiotics | 0=No  1=Yes |
| outcome_ intravenous | The proportion that contain any antibiotics delivered by intravenous injection | 0=No  1=Yes |
| outcome_chinese | The proportion containing any Traditional Chinese Medicines | 0=No  1=Yes |
| price_medicine | The cost of an ARI prescription, based on the cost of any medicines | Continuous variable |
| price_all | The cost of a consultation, based on all costs including medicines, tests and the consultation | Continuous variable |

# Table S1. CRP&SAA levels in those with an initial antibiotic prescription.

| SAA values | CRP values | Treatment options | All patients (n=1311) |
| --- | --- | --- | --- |
| < 100 mg / L | < 10 mg / L | No antibiotics are recommended | 278/940 (30%) |
| < 100 mg / L | 10 mg / L~50 mg / L | No antibiotics are recommended | 82/187 (44%) |
| < 100 mg / L | > 50 mg / L | Antibiotics are recommended | 15/21 (71%) |
| ≥ 100 mg / L | < 10 mg / L | No antibiotics are recommended | 7/17 (41%) |
| ≥ 100 mg / L | 10 mg / L~50 mg / L | Antibiotics are recommended | 41/94 (44%) |
| ≥ 100 mg / L | > 50 mg / L | Antibiotics are recommended | 33/52 (63%) |

Abbreviation: CRP, C-reactive protein; SAA, Serum amyloid A

# Figure S1. Number of initial consultations for ARIs per village clinic over a six-month period.


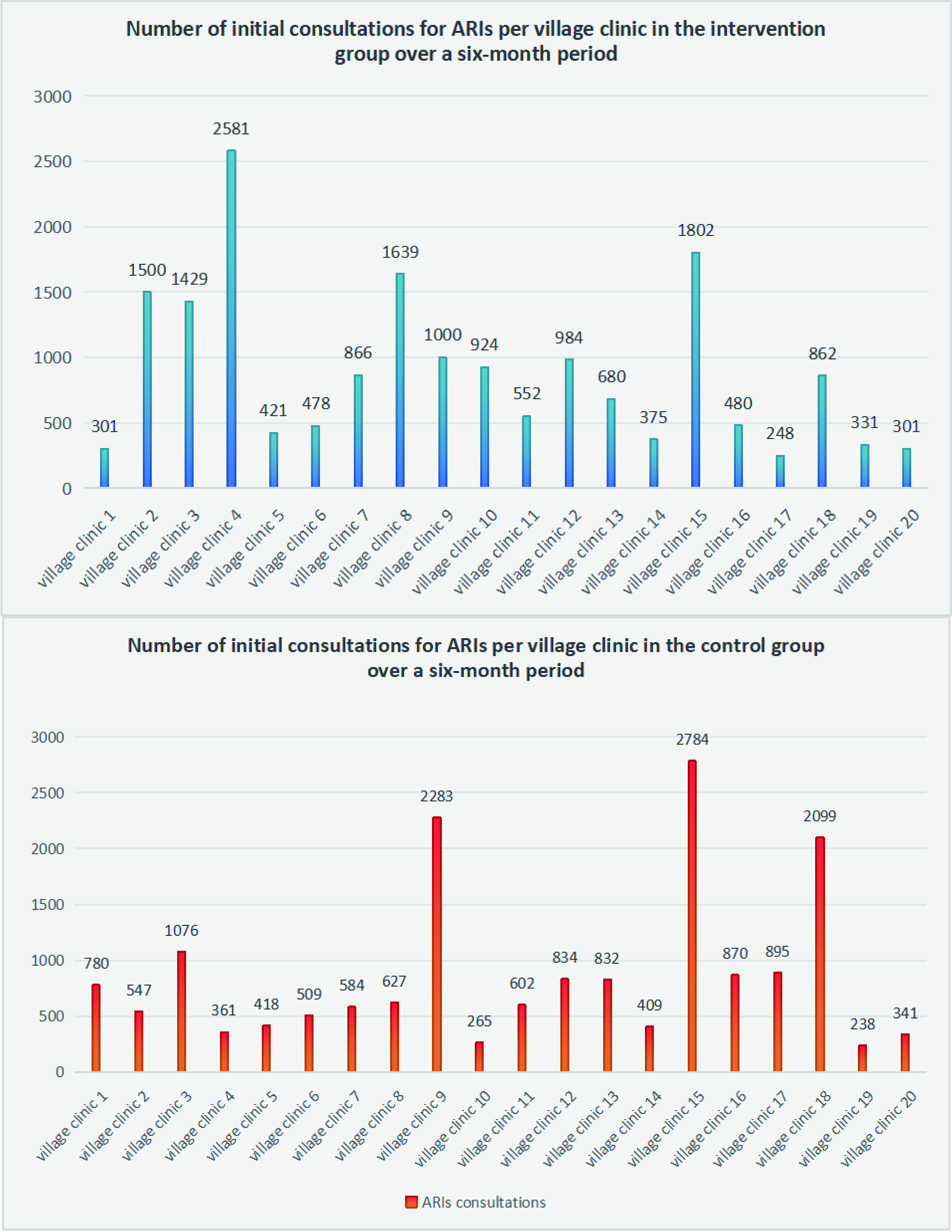


# Figure S2. The frequency of POCT utilisation in the intervention group.


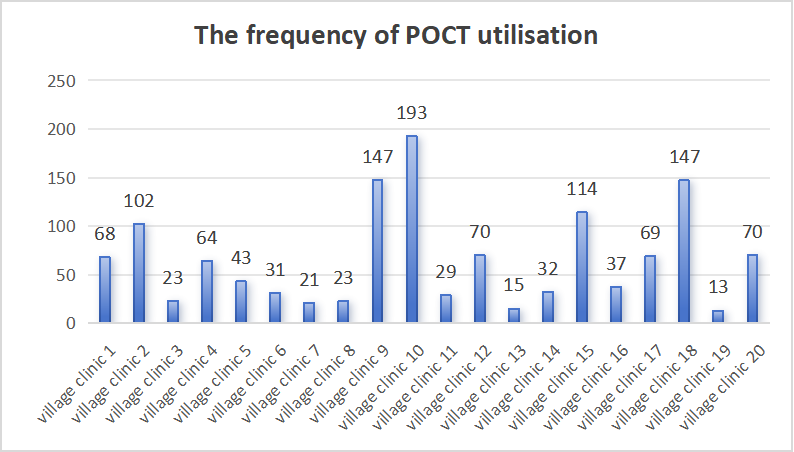


# Table S2. Sensitivity analyses of the comprehensive intervention package based on CRP&SAA POCT effects on outcomes

| **Outcomes** | **Crude relative risk (95% CI)** | **p value** | **Crude risk**  **difference (95% CI)** | **p value** |
| --- | --- | --- | --- | --- |
| **Primary outcome** |  |  |  |  |
| Antibiotic prescription rate |  |  |  |  |
| Intention to treat | 0.77 (0.75 to 0.78) | < 0.001 | -16% (-17 to -14) | < 0.001 |
| Per protocol | 0.53 (0.49 to 0.57) | < 0.001 | -31% (-34 to -28) | < 0.001 |
| **Secondary outcomes** |  |  |  |  |
| Multiple antibiotic prescription rate | 0.30 (0.27 to 0.33) | < 0.001 | -8% (-9 to -7) | < 0.001 |
| Intravenous antibiotic prescription rate | 0.70 (0.67 to 0.72) | < 0.001 | -12% (-13 to -11) | < 0.001 |
| Traditional Chinese medicine prescription rate | 1.05 (1.03 to 1.08) | < 0.001 | 3% (1 to 4) | < 0.001 |
| **Patient safety indicator** |  |  |  |  |
| 30-day hospitalisation for ARI or sepsis after initial visit | 1.11 (0.76 to 1.63) | 0.58 | 0% (0 to 0) | 0.58 |

ARI = acute respiratory infection. Data are n/N (%). Crude relative risks (RR) and risk differences (RD) were estimated using generalised estimating equations. Models included fixed effects for treatment group and township. Analyses were clustered by village clinic to account for within-clinic correlation.

# Table S3. Sensitivity analyses of the comprehensive intervention package based on CRP&SAA POCT effects on prescription costs

| **Cost** | **Crude mean**  **difference (95% CI)** | **p value** |
| --- | --- | --- |
| Full consultation cost (US$)^*^ | -0.06 (-0.11 to -0.01) | 0.01 |
| Medication cost (US$) | 0.03 (-0.01 to 0.08) | 0.17 |

Data are mean (SD). Crude intervention vs control mean difference results were estimated using generalised estimating equations. Models included fixed effects for treatment group and township. Analyses were clustered by village clinic to account for within-clinic correlation. ^*^Full consultation cost includes medicines, treatment, tests and the consultation. US$ values are based on the currency exchange rate on December 17, 2025, in which 1US$=7.05RMB.

# Table S4. Cluster-level analysis of the effects of the comprehensive intervention package based on CRP&SAA POCT on outcomes

| **Outcomes** | **Control group (n=20 clusters)** | **Intervention group (n=20 clusters)** | **Mean difference (95% CI)** | **p value** |
| --- | --- | --- | --- | --- |
| **Primary outcome** |  |  |  |  |
| Antibiotic prescription rate | 63.14% (19.43%) | 53.38% (16.11%) | -17% (-29 to -4) | 0.009 |
| **Secondary outcomes** |  |  |  |  |
| Multiple antibiotic prescription rate | 7.25% (17.20%) | 3.74% (6.93%) | -9% (-21 to 3) | 0.124 |
| Intravenous antibiotic prescription rate | 37.06% (28.13%) | 30.72% (16.57%) | -13% (-29 to 4) | 0.125 |
| Traditional Chinese Medicine prescription rate | 46.28% (21.88%) | 49.89% (18.09%) | 3% (-11 to 17) | 0.681 |

# Cluster-level summary data are mean (SD) of cluster-level outcome percentages.

# Table S5. Cluster-level analysis of the effects of the comprehensive intervention package based on CRP&SAA POCT on prescription costs

| **Cost** | **Control group (n=20 clusters)** | **Intervention group (n=20 clusters)** | **Adjusted mean**  **difference (95% CI)** | **p value** |
| --- | --- | --- | --- | --- |
| Full consultation cost (US$)^*^ | 3.92 (0.82) | 4.01 (0.66) | -0.10 (-0.68 to 0.49) | 0.743 |
| Medication cost (US$) | 2.59 (0.73) | 2.66 (0.64) | 0.02 (-0.51 to 0.55) | 0.933 |

Cluster-level summary data are mean (SD) of cluster-level outcome means. ^*^Full consultation cost includes medicines, treatment, tests and the consultation. US$ values are based on the currency exchange rate on December 17, 2025, in which 1US$=7.05RMB.

# Table S6. Effectiveness of the comprehensive intervention package based on CRP&SAA POCT on outcomes: using generalised estimating equations with delete-one-cluster Jackknife correction to address potential small-sample bias

| **Outcomes** | **Control group (n=20 clusters)** | **Intervention group (n=20 clusters)** | **Adjusted relative risk (95% CI)** | **p value** | **Adjusted risk**  **difference (95% CI)** | **p value** |
| --- | --- | --- | --- | --- | --- | --- |
| **Primary outcome** |  |  |  |  |  |  |
| Antibiotic prescription rate |  |  |  |  |  |  |
| Intention to treat | 11715/17354 (67.51%) | 9001/17754 (50.70%) | 0.80 (0.78 to 0.82) | < 0.001 | -13% (-15 to -12) | < 0.001 |
| Per protocol | 11715/17354 (67.51%) | 456/1311 (34.78%) | 0.52 (0.46 to 0.58) | < 0.001 | -29% (-33 to -24) | < 0.001 |
| **Secondary outcomes** |  |  |  |  |  |  |
| Multiple antibiotic prescription rate | 2163/17354 (12.46%) | 584/17754 (3.29%) | 0.40 (0.35 to 0.46) | < 0.001 | -6% (-7 to -5) | < 0.001 |
| Intravenous antibiotic prescription rate | 6874/17354 (39.61%) | 4785/17754 (26.95%) | 0.69 (0.67 to 0.72) | < 0.001 | -13% (-15 to -12) | < 0.001 |
| Traditional Chinese Medicine prescription rate | 8301/17354 (47.83%) | 9013/17754 (50.77%) | 1.08 (1.04 to 1.11) | < 0.001 | 3% (2 to 5) | < 0.001 |
| **Patient safety indicator** |  |  |  |  |  |  |
| 30-day hospitalisation for ARI or sepsis after initial visit | 55/17354 (0.32%) | 63/17754 (0.35%) | 0.94 (0.59 to 1.48) | 0.78 | 0% (0 to 0) | 0.78 |

ARI = acute respiratory infection. Data are n/N (%). Adjusted relative risks (RR) and risk differences (RD) were estimated using generalised estimating equations with delete-one-cluster Jackknife correction. Models included fixed effects for treatment group, village doctor characteristics (sex, age, education level), patient characteristics (age, sex, type of acute respiratory infection), and township. Analyses were clustered by village clinic to account for within-clinic correlation.

# Table S7. Effect of the comprehensive intervention package based on CRP&SAA POCT on prescription costs: using generalised estimating equations with delete-one-cluster Jackknife correction to address potential small-sample bias

| **Cost** | **Control group (n=20 clusters)** | **Intervention group (n=20 clusters)** | **Adjusted mean**  **difference (95% CI)** | **p value** |
| --- | --- | --- | --- | --- |
| **Intention to treat** |  |  |  |  |
| Full consultation cost (US$)^*^ | 4.06 (1.97) | 3.96 (2.14) | -0.04 (-0.08 to -0.01) | 0.02 |
| Medication cost (US$) | 2.64 (1.85) | 2.66 (2.14) | -0.01 (-0.05 to 0.02) | 0.46 |
| **Per protocol** |  |  |  |  |
| Full consultation cost (US$)^*^ | 4.06 (1.97) | 4.65 (1.79) | 0.79 (0.67 to 0.91) | < 0.001 |
| Medication cost (US$) | 2.64 (1.85) | 2.58 (1.66) | -0.02 (-0.14 to 0.09) | 0.71 |

Data are mean (SD). Adjusted intervention vs control mean difference results were estimated using generalised estimating equations with delete-one-cluster Jackknife correction. Models included fixed effects for treatment group, village doctor characteristics (sex, age, education level), patient characteristics (age, sex, type of acute respiratory infection), and township. Analyses were clustered by village clinic to account for within-clinic correlation. ^*^Full consultation cost includes medicines, treatment, tests and the consultation. US$ values are based on the currency exchange rate on December 17, 2025, in which 1US$=7.05RMB.

**Table S8**: Post-hoc 1:1 propensity score-matched analysis of the primary outcome, stratified by ARI type and patient age (Intervention cases receiving POCT vs. Controls)

| **Outcomes** | **Control group (n=20 clusters)** | **Intervention group (n=20 clusters)** | **Adjusted relative risk (95% CI)** | **p value** | **Adjusted risk**  **difference (95% CI)** | **p value** |
| --- | --- | --- | --- | --- | --- | --- |
| **Primary outcome** |  |  |  |  |  |  |
| Antibiotic prescription rate | 864/1311 (65.90%) | 456/1311 (34.78%) | 0.53 (0.46 to 0.60) | < 0.001 | -28% (-32 to -23) | < 0.001 |

ARI = acute respiratory infection. Data are n/N (%). Adjusted relative risks (RR) and risk differences (RD) were estimated using generalised estimating equations. Models included fixed effects for treatment group, village doctor characteristics (sex, age, education level), patient characteristics (age, sex, type of acute respiratory infection), and township. Analyses were clustered by village clinic to account for within-clinic correlation.
