# Supplementary material for: Effect of a comprehensive package based on combined C-reactive protein and serum amyloid A point-of-care testing on antibiotic prescribing for acute respiratory tract infections at village clinics in China: a cluster randomised controlled trial
Source: Lancet Reg Health West Pac. 2026 May 25;71:101888. doi: 10.1016/j.lanwpc.2026.101888 (PMC13227237; doi:10.1016/j.lanwpc.2026.101888)
Supplement: Protocol [file mmc2.docx]

**STUDY PROTOCOL**

**Effect of a comprehensive package based on combined C-reactive protein and serum amyloid A point-of-care testing on antibiotic prescribing for acute respiratory tract infections at village clinics in China**

Minzhi Xu^1^, Zhitong Zhang^2^, Erjia Ge^2^, Charis Xuan Xie^3^,Xinyu Bai^1^, Yuting Zhu^1^, Guangjin Kuang^1^, Jinxi Li^1^, Jing Wang^1^, Xiaolin Wei^2^, Xiaoxv Yin^1^

**Affiliation Information**

^1^Department of Social Medicine and Health Management, School of Public Health, Tongji Medical College, Huazhong University of Science and Technology, Wuhan, Hubei, China

^2^Dalla Lana School of Public Health, University of Toronto, Toronto, ON, Canada

^3^Wolfson Institute of Population Health, Queen Mary University of London, England, UK

[1. Introduction 2](#_Toc30918)

[2. Material and methods 3](#_Toc54)

[3. Study design 4](#_Toc3627)

[4. Setting 5](#_Toc9266)

[5. Eligibility 6](#_Toc23643)

[6. Recruitment timeline 6](#_Toc22354)

[7. Ethical considerations and dissemination 6](#_Toc1412)

[8. Intervention 7](#_Toc16735)

[9. Usual care 10](#_Toc3946)

[10. Allocation and blinding 10](#_Toc24990)

[11. Outcomes 11](#_Toc12918)

[12. Sample size 12](#_Toc13380)

[13. Internal pilot process 12](#_Toc12740)

[14. Data collection and management 12](#_Toc20662)

[15. Data analysis plan 13](#_Toc3324)

[16. Process evaluation 14](#_Toc11910)

[17. Trial management 14](#_Toc8651)

[18. Discussion 14](#_Toc28549)

[19. Reference 15](#_Toc16937)

# Introduction

Antimicrobial resistance is a globally recognised public health threat, and one of its primary drivers is the significant increase in antibiotic prescriptions.^1^ In China, the use of antibiotics is common in rural areas.^2,3^ Existing studies indicate that the usage of antibiotics in village clinics approaches or exceeds 50%, which is significantly higher than the rates observed in other levels of healthcare.^3,4^ Acute respiratory infections (ARIs), which include symptoms such as coughing and fever, are among the primary reasons for antibiotic prescriptions in village clinics.^5^ However, only a small proportion of patients with bacterial ARIs actually require antibiotic treatment.^6,7^ These unnecessary prescriptions can lead to adverse reactions, incur excessive costs, and increase overall antibiotic consumption. Identifying effective interventions to reduce the inappropriate use of antibiotics for ARIs in village clinics could significantly improve antibiotic use in China.

It is challenging to accurately differentiate between bacterial and viral ARIs due to the overlapping symptoms of both types. This similarity complicates the diagnostic process and is a significant factor contributing to the irrational use of antibiotics for treating ARIs.^7,8^ In recent years, certain biomarkers, such as C-reactive protein (CRP), have increasingly been utilised to inform antibiotic prescribing practices for ARIs.^8–10^ A clinical trial conducted in Vietnam demonstrated that CRP point-of-care testing (POCT) in primary care settings effectively reduced antibiotic usage among patients with ARIs, without impacting the duration of symptoms between the two groups.^11^ Another clinical trial in Thailand and Myanmar involving patients with fever, the majority of whom exhibited respiratory symptoms, yielded similar results.^12^ However, several randomised controlled trials conducted in high-income countries have indicated that the use of CRP POCT alone does not reduce antibiotic prescribing for ARIs in primary care settings.^7,13^ Although CRP POCT is a valuable tool in diagnosing bacterial infections, its specificity is limited, as viral infections can also lead to elevated CRP levels. Additionally, since CRP is part of a dynamic and continuous inflammatory process, a single CRP measurement, especially at low concentrations, may erroneously classify an infection as viral rather than bacterial, potentially delaying appropriate antibiotic treatment.^14^ The 2022 Cochrane review concluded that further research is necessary to evaluate the effectiveness of new potential biomarkers to guide antibiotic prescribing in primary care.^15^ The clinical significance of serum amyloid A (SAA) is promising as changes in its levels play a crucial role in the early diagnosis of infectious diseases.^16–18^ SAA demonstrates high diagnostic accuracy for viral infections and is significantly elevated during the acute phase of such infections.^19^ By assessing the degree of SAA and combining it with CRP, it is possible to differentiate between bacterial and viral infections more accurately. This indication of a combined biomarker addresses the limitations of currently used single biomarkers, which often fail to indicate viral infections specifically.

Despite its significant potential, the evidence supporting the use of CRP&SAA POCT for antibiotic management of ARIs in primary care settings remains insufficient. The simplicity, rapidity, low cost, and high accuracy of CRP&SAA POCT make it particularly suitable for remote primary healthcare settings, such as village clinics in China. Therefore, in this study, we aim to evaluate the effectiveness of a comprehensive intervention based on CRP&SAA POCT, which we hypothesised would reduce the inappropriate use of antibiotics for ARIs in Chinese village clinics.

# Material and methods

We used the SPIRIT (Standard Protocol Items: Recommendations for Interventional Trials) reporting guidelines for clinical trials.^20^ The timeline for enrollment, interventions, and assessments are presented in **Figure 1**.

**Figure 1** SPIRIT schedule of enrolment, interventions, and assessments.


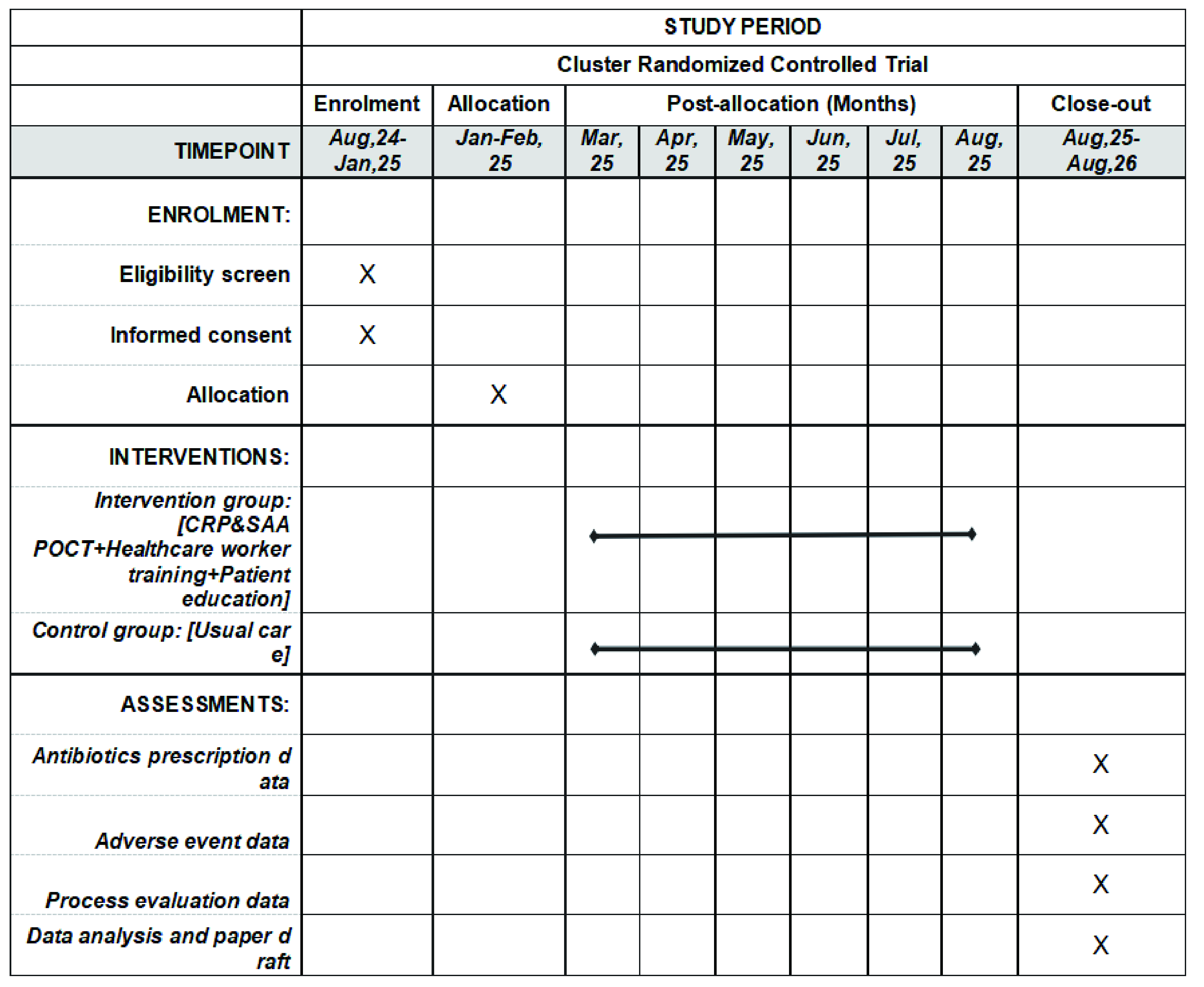


# Study design

This is a pragmatic, parallel-group, cluster randomised controlled trial designed to evaluate the impact of a CRP&SAA POCT-based comprehensive intervention on antibiotic prescribing for patients with ARIs in Chinese village clinics (**Figure 2**). This study will be conducted over a period of six months across 40 village clinics, with randomization stratified by township. CRP&SAA POCT will be implemented in 20 village clinics within the intervention arm. This will include additional training for village doctors on the operations of CRP&SAA POCT, which encompasses centralised training, the distribution of training manuals, and desk reminders. The aim is to help village doctors understand the significance of CRP&SAA POCT in guiding antibiotic prescriptions and to familiarise them with the specific operational procedures. Additionally, patient education materials will be provided to assist patients in understanding how CRP&SAA POCT can aid in their diagnosis and treatment. The control arm will not receive any intervention and will continue with usual care.

**Figure 2** Trial flow chart.


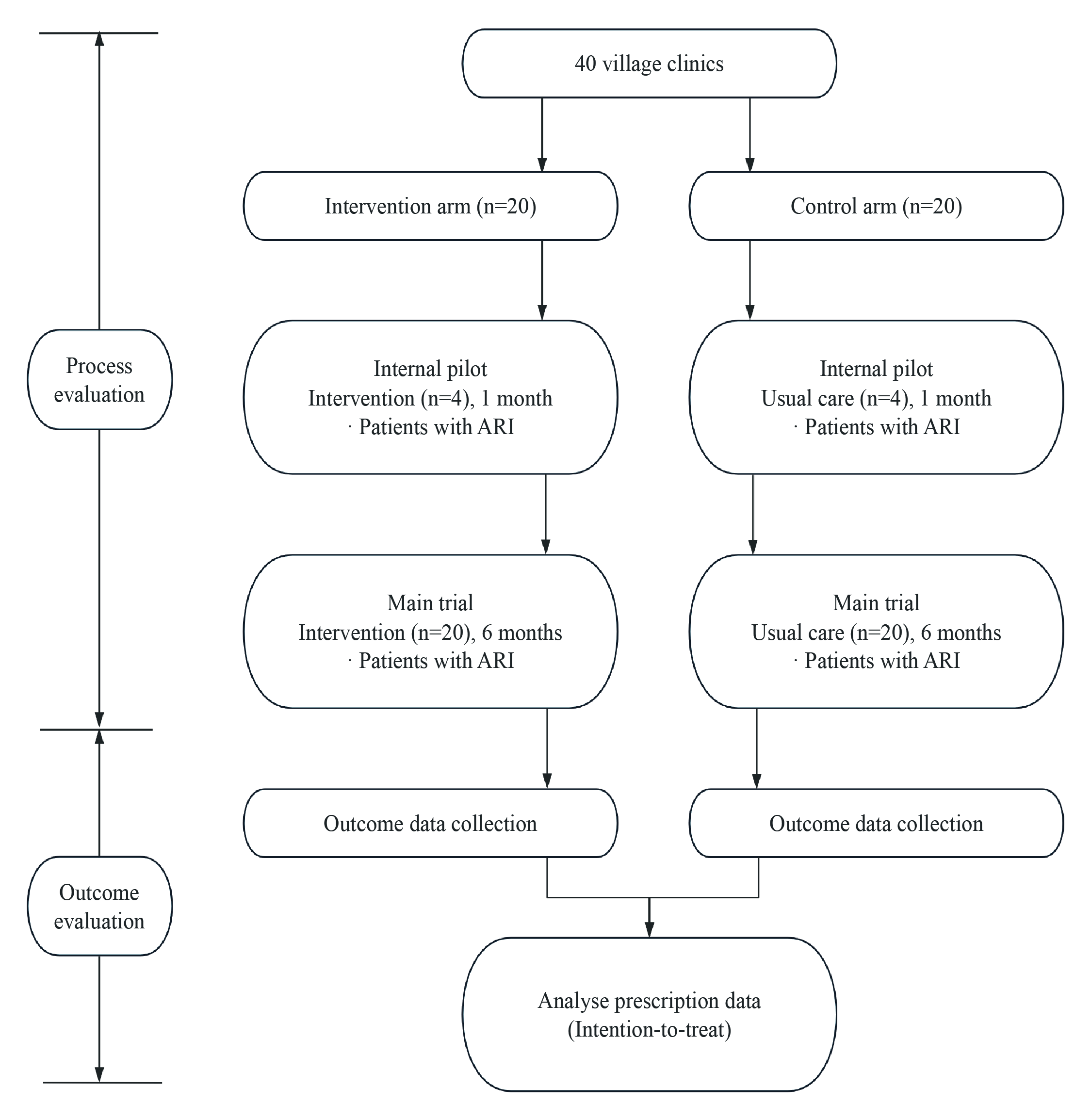


# Setting

As the primary providers of health care services in rural China, village clinics address various aspects, including basic medical services, preventive health care, public health services, and drug supply, with the goal of meeting the fundamental medical and public health needs of rural residents. This study will be conducted in 40 village clinics across 15 townships and 4 sub-district offices (equivalent to townships) in Xiantao, Hubei Province. Xiantao is situated in central China’s Hubei Province, with a population of approximately 1.11 million permanent residents. The city administers 15 townships and 4 sub-district offices. Each village clinic employs 1 to 2 upgraded community health workers, commonly referred to as ‘village doctors’. All village doctors have successfully passed the national doctor qualification examination and have obtained either a certificate of practicing physician qualification or a certificate of practicing assistant physician qualification. This includes qualifications for practicing as a physician or assistant physician in the clinical category, as well as the rural general practice assistant physician qualification.

# Eligibility

*5.1 Eligibility criteria for clusters*

Village clinics with an annual outpatient volume exceeding 2000, an average of 10 or more patients per week presenting with ARIs, and licensed prescribers are considered eligible for selection for the intervention. Annual outpatient prescriptions and average weekly visits for ARIs will be verified by obtaining prescription data from all village clinics for the previous year, as documented in the information section of the local health board.

*5.2 Eligibility criteria for participants*

The target population of this study included (1) patients of all ages diagnosed by a village doctor with ARIs (including upper and lower respiratory infections); and (2) patients who present with ≥1 acute respiratory symptoms (including cough, rhinitis (sneezing, nasal congestion or runny nose), sore throat, shortness of breath, wheezing or abnormal auscultation). Patients with non-respiratory diseases or those with severe clinical symptoms requiring referral to a higher-level institution are excluded from the target population.

# Recruitment timeline

Participant recruitment began on 15 February 2025, and the intervention will run for six months from 15 February 2025 to 15 August 2025. Currently (March 2025), the intervention is ongoing, and the study has not yet generated results. Participant recruitment will continue throughout the intervention period (until 15 August 2025).

The pilot work has started under the local ethical approval and has finished by the time of submission. Reporting of study results is expected before August 2026. The total lifespan of the study will be 24 months.

# Ethical considerations and dissemination

The trial has obtained ethical approval from the ethics committee of Huazhong University of Science and Technology (Ref: S129). A waiver of written informed consent from patients (or their legally authorized representatives) to participate in the study was obtained from the relevant ethical review boards to minimize disruption to routine practice. The trial was registered at ClinicalTrials.gov: NCT06568432 on 21 August 2024 (https://clinicaltrials.gov/study/NCT06568432).

This initiative is supported by funding from the University of Toronto-Huazhong University of Science and Technology Joint Seed Fund. Throughout the research project, we will implement a CRP&SAA POCT-based comprehensive intervention in village clinics across China to improve antibiotic use in ARIs. We will produce policy briefs and recommendations to influence current practices in primary healthcare institutions. Additionally, the findings will be published in internationally peer-reviewed journals through peer-reviewed publications and presented at academic conferences in China, Canada, and other countries.

# Intervention

According to national and international guidelines, the self-limiting virus ARIs should not be treated with antibiotics. The intervention aimed to guide the prescribing behavior of ARIs by doctors in Chinese village clinics through the use of CRP&SAA POCT. The purpose and design of this study adhered to policy requirements for antibiotic prescribing and are subject to routine oversight by local health authorities. To facilitate the implementation of CRP&SAA POCT, the intervention design is guided by the Theoretical Domain Framework (TDF), an emerging approach developed from a diverse array of theories related to behavior change.^21^ The TDF comprises 14 theoretical domains and is widely utilised in implementation studies to design interventions and examine factors that influence their effectiveness.^22,23^ Several theoretical areas within the TDF were identified as crucial for CRP&SAA POCT-guided antibiotic prescribing, where the content of relevant behavior change techniques and interventions was specifically targeted (**Table 1**).

**Table 1** A multidimensional intervention utilizing CRP&SAA POCT aimed at reducing antibiotic use in patients with ARIs in village clinics.

| **Target population** | **Theoretical domains** | **Behaviour change techniques, modes and content of delivery** |
| --- | --- | --- |
| Village doctors | Knowledge  Skills | Techniques: information provision  Mode 1: centralised training.  Content: (1) the operational process for CRP&SAA POCT; (2) recommended thresholds for CRP&SAA, along with practical case applications; and (3) clinical evaluation and management of ARIs.  Mode 2: guidance manual.  Content: (1) an introduction to ARIs; (2) standardised procedures for the diagnosis and treatment of ARIs; (3) clinical diagnosis and treatment pathways for ARIs based on CRP&SAA POCT; (4) a brief overview of the principles of CRP&SAA POCT, along with illustrated operational steps.  Mode 3: desktop reminders.  Content: the recommended thresholds from CRP&SAA |
|  |  |  |
|  | Reinforcement | Techniques: incentive.  Mode: charged for CRP&SAA POCT  Contents: each patient with an ARI utilizing the CPR&SAA POCT will ultimately pay 5 yuan (approximately US $0.68) to the village clinic. |
|  | Behaviour regulation | Techniques: feedback and monitoring.  Mode: appraisal of antibiotic prescribing  Contents: (1) at the beginning of each month following the intervention, the research team will collect prescription information for ARIs from all village clinics within the intervention arm; (2) conduct a comprehensive assessment to determine whether village doctors are prescribing antibiotics in accordance with the CRP&SAA POCT threshold; (3) provide feedback to the relevant village clinics and administrative staff. |
| ARI patients | Knowledge  Beliefs about consequences | Techniques: information provision.  Mode: health education  Content: a popular science leaflet outlines the role of CRP&SAA POCT in promoting the rational use of antibiotics for ARIs, as well as the risks associated with antibiotic overuse. |
|  |  |  |

Abbreviation:CRP, C-reactive protein; SAA, Serum amyloid A; POCT, point-of-care testing; ARIs, acute respiratory infections.

*8.1 CRP&SAA POCT*

Serum Amyloid A/C-Reactive Protein Combined Assay Kit (Immunochromatography) will be supplied to all village clinics in the intervention arm for the target population (ARIs patients). Since the purpose of this study is to determine whether antibiotic prescribing for ARIs can be guided by the use of CRP&SAA POCT, the intervention arm should be encouraged to utilise CRP&SAA POCT for all target populations (patients with ARIs). This recommendation is suggested but not mandated. The control arm will not receive any intervention (usual care).

Village doctors will decide whether to prescribe antibiotics to patients with ARIs based on the recommended threshold for CRP&SAA levels (mg/L) (**Table 2**). As there is currently no specific cut-off threshold for CRP and SAA, our recommended threshold was developed in conjunction with previous studies and expert consensus in China to align more closely with clinical practice.^7,9,24^ In order to further encourage village doctors to utilise CRP&SAA POCT, we simulated real-world conditions. According to the reimbursement policy, the Rural Cooperative Medical Care in China, provides a 60% reimbursement for visits to village clinics. Each patient with an ARI utilizing the CPR&SAA POCT, originally priced at 14 yuan (approximately US $1.92), will ultimately pay 5 yuan (approximately US $0.68) to the village clinic.

**Table 2** Guidance available to village doctors on the cut off points used for CRP&SAA values and the relevant treatment options.

| SAA values | CRP values | Treatment options |
| --- | --- | --- |
| < 100 mg / L | < 10 mg / L | No antibiotics are recommended |
| < 100 mg / L | 10 mg / L~50 mg / L | No antibiotics are recommended |
| > 100 mg / L | < 10 mg / L | No antibiotics are recommended |
| < 100 mg / L | > 50 mg / L | antibiotics are recommended |
| > 100 mg / L | 10 mg / L~50 mg / L | antibiotics are recommended |
| > 100 mg / L | > 50 mg / L | antibiotics are recommended |

Abbreviation: CRP, C-reactive protein; SAA, Serum amyloid A

*8.2 Healthcare worker training*

After randomization, we will implement a unified and centralised training program for doctors in all village clinics within the intervention arm. The training will cover the following topics: (1) the operation process for CRP&SAA POCT; (2) recommended thresholds for CRP&SAA, along with practical case applications; and (3) clinical evaluation and management of ARIs. Following the intensive training, the research team will provide on-site guidance for the use of CRP&SAA POCT in each village clinic within the intervention arm for one week. This support will ensure that each village doctor is capable of independently operating the CRP&SAA POCT. Training will be conducted by the research team, and an online communication group will be established to address any questions that village doctors may have during the follow-up process.

The research team will distribute a CRP&SAA POCT-based village clinic guidance manual on the rational use of antibiotics for ARIs to each village clinic in the intervention arm. This guideline includes an introduction to ARIs, standardised procedures for their diagnosis and treatment, CRP&SAA POCT-based clinical diagnosis and treatment pathways for ARIs, a brief overview of the principles of CRP&SAA POCT, and illustrated operational steps. Desktop reminders featuring the recommended thresholds from CRP&SAA will be distributed to the intervention arm. Village doctors in the intervention arm will be informed that the CRP&SAA recommended threshold serves only as a suggestion, and that antibiotic prescribing must ultimately be determined based on the clinical judgment. If a village doctor believes that patients with ARIs require referral to a higher level of care, the referral should be done immediately, and CRP&SAA POCT should not be utilised.

At the beginning of each month following the intervention, the research team will gather prescription information for ARIs from all village clinics within the intervention arm. The team will conduct a comprehensive assessment to determine whether village doctors are prescribing antibiotics in accordance with the CRP&SAA POCT threshold. Subsequently, the team will provide feedback to the relevant village clinics and administrative staff.

*8.3 Patient education*

For the village clinics in the intervention arm, patients presenting with ARIs will receive a popular science leaflet detailing the role of CRP&SAA POCT in promoting the rational use of antibiotics for ARIs, as well as the risks associated with antibiotic overuse. According to the information provided in the publicity leaflet and the advice of the village doctor, patients with ARIs (or their legally appointed agents) will determine whether to consent to CRP&SAA POCT based on their individual circumstances.

# Usual care

Village clinics in the control arm will continue to prescribe antibiotics to patients with ARIs in accordance with the current national guidelines and usual care in village clinics. In usual care, village doctor will diagnose and treatment of ARIs based on their clinical experience and existing knowledge, and make decision on whether to prescribe antibiotics. Notably, the village doctors in the control arm will not receive any education on ARIs and the appropriate use of antibiotics, and will not receive the Serum Amyloid A/C-Reactive Protein Combined Assay Kit.

# Allocation and blinding

The 40 village clinics will be allocated to either the intervention or control arm using stratified permuted block randomization, stratified by township to ensure balanced distribution across regions and account for potential variation in outcomes between townships. Randomisation will be conducted using the *carat* package in R version 4.0.5 (R Foundation for Statistical Computing). ARIs patients and village doctors will not be blinded to the treatment; however, measures will be implemented to ensure a blinded outcome evaluation by employing the ‘PROBE’ design.^25^ Analysts should remain blinded during the analysis.

# Outcomes

*11.1 Primary outcome*

The primary outcome is the proportion of patients who are diagnosed with ARIs and prescribed antibiotics during their initial visit (defined as no prescription record at the current institution within the preceding 14 days) in both study arms. This outcome serves as the primary indicator, reflecting the overall impact of a comprehensive intervention based on CRP&SAA POCT in guiding antibiotic use for patients with ARIs. Since most self-limiting ARIs are caused by viral infections that do not require antibiotic treatment, the decline in antibiotic prescribing rates suggests that village doctors are prescribing antibiotics more judiciously.^23^ The selection of this outcome is both feasible and reliable within the context of village clinics in China. This is due to the transition of prescriptions from traditional paper documents to electronic storage, which allows for the proper preservation of prescription records, thereby ensuring data integrity and traceability.

*11.2 Secondary outcomes*

Secondary outcomes will be extracted from the prescribing information related to the primary outcome, as well as from subsequent visit information. In particular, we included the proportion of participants using any form of Traditional Chinese Medicines as a secondary outcome. In our previous trials, we observed an increase in the use of Traditional Chinese Medicines, possibly as an alternative to antibiotics.^23^ These outcomes will include the following types:

1. The proportion of multiple antibiotic prescriptions in the intervention and control arms (specifically, the proportion of ARI prescriptions that include two or more antibiotics).
2. The intravenously injected antibiotic prescription rate (the proportion that contain any antibiotics delivered by intravenous injection).
3. The proportion containing any Traditional Chinese Medicines.
4. The mean cost of an ARI prescription, based on the cost of any medicines.
5. The mean cost of a consultation, based on all costs including medicines, tests and the consultation.

*11.3 Patient safety indicator*

We will evaluate whether the intervention appears to increase the incidence of adverse events. This may occur if antibiotics are more frequently withheld for appropriate conditions as a result of the intervention. To address this concern, we will use the proportion of patients with ARIs in both the intervention and control arms who are hospitalised in Xiantao City for ARIs or sepsis within 30 days following their initial ARIs visit, excluding those who are referred by village doctors during their initial consultations, as a safety indicator.

# Sample size

Based on our exploratory study and previous research, the antibiotic prescription rate in rural primary healthcare institutions in China ranges from 50% to 80%.^3,4,23^ The minimum value of 50% is utilised as a conservative estimate of the conventional antibiotic prescription rate in the control arms. We conservatively estimate that CRP&SAA POCT intervention will lead to at least a 25% relative reduction in the antibiotic prescription rate in village clinics. Thus, to detect a reduction of 25% or more in the antibiotic prescription rate - specifically, an absolute reduction to 37.5% antibiotic prescription rate or lower - with 90% power, utilizing two-sided testing at the 5% significant level, and assuming a harmonic mean cluster size of 500 along with an intracluster correlation coefficient of 0.05, we estimate that we will require 17 village clinics per arm.^26^ Allowing for a 15% loss of prescription data and considering that 20 village clinics are required per arm, it was decided to include a total of 40 village clinics.

# Internal pilot process

Before initiating the main trial, we will evaluate the feasibility of implementing the intervention through an internal pilot process. During the preparation period, 8 village clinics (from 4 townships) will be selected as pilot sites and randomly assigned to either the intervention or control arm. They will be followed up for one month to evaluate the feasibility of the following objectives: (1) whether all village doctors in the intervention arm can independently operate the CRP&SAA POCT; and (2) whether the doctors can effectively guide the antibiotic prescription for patients with ARIs based on the varying cut-off thresholds of the CRP&SAA POCT. We will adjust the implementation plan as necessary based on the insights gained from the internal pilot process.

# Data collection and management

Upon the commencement of the trial, prescription information for ARI patients from all enrolled village clinics will be collected within a six-month period using a routine electronic health record system. Each ARI patient will be identified by their ID number. The information section of the local health board will connect the electronic health record system of the village clinics with all hospitals above the village clinics level in Xiantao to facilitate the identification of hospitalizations. Finally, the local health board will employ a specific algorithm to generate a unique study number for each patient based on their ID number. This algorithm will not disclose information to the study investigators or data analysts. The generated study numbers will be used to create an electronic study database, ensuring the protection of patient privacy. Patient names and detailed addresses will not be included in the electronic research database; all information collected will be encrypted. All data will be stored on secure computers that are accessible only to authorised data managers.

# Data analysis plan

All analyses will be conducted on the intention-to-treat (ITT) population, defined as all outpatient prescriptions issued by village clinics for ARI patients, regardless of the compliance of village doctors and patients with the intervention. For the primary and secondary outcomes, which are binary variables, we will employ Generalised estimating equations (GEEs) to estimate both relative and absolute measures of effect.^27^ Specifically, a GEE with binomial errors and an identity link will be employed to estimate the absolute difference in proportions (intervention minus control) of all binary outcomes between the intervention and control arms. A Poisson GEE with log link and robust variance estimator will be applied to estimate relative risks (RR). For continuous outcomes, such as the mean cost of an ARI prescription and consultation fee, we will use linear regression with GEE, again accounting for within-clinic correlation. We will assume an exchangeable correlation structure at the village clinic level to account for within-cluster correlation, and estimate parameter standard errors using the Huber-White (robust) estimator. Village doctors’ characteristics (sex, age, and qualification level), ARI patient’s characteristics (age, sex, type of ARI), and township will be adjusted in models. We will base our principle inference about the effectiveness of the intervention on this main analysis. We expect minimal missing data due to the use of routinely collected electronic records. If missing data are less than 5%, a complete-case analysis will be performed. If missingness exceeds 5%, multiple imputation using chained equations (MICE) will be conducted under the assumption of missing at random, incorporating key baseline and outcome variables into the imputation model.

The primary outcome will also be analysed in subgroups based on ARI patient’s age (1-15 / 16-65 / over 65 years old), ARI patient’s sex (male / female), type of ARI (upper respiratory tract infection / lower respiratory tract infection), and season. Likelihood ratio tests will be employed to assess the heterogeneity of antibiotic prescribing across these different subgroups. We also will conduct per-protocol analyses, which included only ARI patients who received CRP&SAA POCT in village clinics within the intervention arm. To explore the robustness of our results, we will conduct covariate-sensitivity analyses for all outcomes, excluding all covariates except for the stratum. This approach will allow us to examine the stability of the treatment effect when not adjusting for potentially imbalanced covariates.

We will claim statistical significance at the 5% level, and base our inferences on the two-sided p values and associated 95% confidence intervals of the treatment effect estimates. No interim analysis is planned during the course of the study, as the trial involves no more than minimal risk to participants. Given that the intervention has previously been demonstrated to be safe, we do not intend to implement stopping rules.^12^

# Process evaluation

A process evaluation will be conducted for all village clinics in the intervention arm. Our specific objectives for the process evaluation are: (1) to describe the health system and service delivery context in which the intervention was implemented; (2) to assess intervention fidelity; (3) to identify the barriers and facilitators in the implementation process and determine which mechanisms influence behavior change outcomes. Specific research methods included prescription reviews, treatment process observations, and qualitative interviews. If the village doctor consents to the use of the recording, qualitative data will be collected. Audio files will be transcribed promptly, and any audio files recorded using a mobile recording device will be deleted immediately after transcription. The process evaluation of this study may serve as a foundation for planning similar projects in other low- and middle-income countries in the future. Our analysis will be based on the TDF while also allowing for the generalization of important newly discovered topics. The steps of our analysis include familiarizing ourselves with the text, reaching a consensus on the coding framework, indexing the content according to the framework, generalizing the findings, and interpreting the results. Nvivo Version 10 (QSR International Pty Ltd) will be utilised to manage the data. The quality of reporting in the qualitative study will be ensured by adhering to the Consolidated Criteria for Reporting Qualitative Research (COREQ).^28^

# Trial management

Prof. Xiaoxv Yin from the Huazhong University of Science and Technology and Prof. Xiaolin Wei from the University of Toronto will be the co-guarantees of the trial and will have full access to the trial dataset. We have established an independent research team composed of the project leader, representatives from the information section of the local health board, and members involved in the specific implementation project. This team will design and monitor the trial, review its progress, and determine any necessary changes to the protocol.

# Discussion

Our study is one of the first trials utilizing CRP&SAA POCT to address the inappropriate prescription of antibiotics for ARIs in village clinics in China. The study aligns with the current national priority of antibiotic control in China. ARIs are the leading cause of often unnecessary antibiotic prescriptions in primary care settings, particularly in developing countries where regulations on antibiotic prescribing are less stringent.^5^ Our study will provide essential diagnostic and treatment information to assist village doctors in making etiological diagnoses. This will not only address the critical issue of the weak specificity of symptoms associated with ARIs but also compensate for the current limitations in the diagnostic and treatment capabilities of village doctors. Additionally, this initiative will help village doctors persuade patients with viral ARIs to avoid antibiotics by providing quantitative evidence. This is particularly important as many rural patients with ARIs have limited awareness of antimicrobials and the risks associated with their misuse, often leading them to request prescriptions for these medications from their doctors. The study is also designed to fit into the rural health insurance scheme. Although previous research has demonstrated the effectiveness of POCT in various national contexts^9,11,12^, a significant gap remains in understanding how to effectively promote the implementation of POCT in practice. This is particularly true in rural areas, where reimbursement rates and out-of-pocket costs for patients are critical factors influencing their willingness to accept POCT, as well as the enthusiasm of village doctors. The implementation of this study will provide a foundation for advocating the inclusion of POCT in the rural health insurance scheme. If successful, it will be poised for adoption into relevant policies. The project is co-led by Huazhong University of Science and Technology and the University of Toronto. A research team has been established to develop and refine the research protocol and to implement the specific trial procedures.

Our trial will face some challenges. First, ARI patients and village doctors will not be blinded to the treatment, which may introduce biases such as the Hawthorne effect.^29^ However, the review of prescriptions and the collection of outcome data during the intervention will be conducted through the electronic medical record system in the information section of the local health department, ensuring minimal interference. Second, a previous study demonstrated a low uptake of POCT interventions in primary care settings.^9^ Promoting the implementation of POCT within the settings of specific national health systems and cultural characteristics presents a significant challenge. The trial aims to incorporate cutting-edge principles of implementation science, including the exploration of barriers and facilitators related to the implementation of CRP&SAA POCT in rural China. It also seeks to evaluate the implementation process to better integrate POCT into local cultural and health system practices, while providing valuable information for scaling up in developing countries that face similar challenges. Third, as a safety endpoint, we will assess the hospitalization rates of patients with ARIs within 14 days of their initial visit to the village clinics. While we successfully linked to the electronic health record systems of hospitals above the village clinic level throughout Xiantao City, data on hospitalisations outside of Xiantao were unavailable, which may lead to an underestimation of hospitalization rates. However, given that our study population consists of patients with ARIs, the diagnostic and treatment technologies at local comprehensive hospitals are well-established. Consequently, the likelihood of hospitalization in other cities is minimal, which will have a negligible impact on our results.

# Reference

1. Butler CC, Hood K, Verheij T, et al. Variation in antibiotic prescribing and its impact on recovery in patients with acute cough in primary care: prospective study in 13 countries. *BMJ*. 2009;338(jun23 2):b2242-b2242. doi:10.1136/bmj.b2242

2. Ding L, Sun Q, Sun W, et al. Antibiotic use in rural China: a cross-sectional survey of knowledge, attitudes and self-reported practices among caregivers in Shandong province. *BMC Infect Dis*. 2015;15(1):576. doi:10.1186/s12879-015-1323-z

3. Wang J, Wang P, Wang X, Zheng Y, Xiao Y. Use and Prescription of Antibiotics in Primary Health Care Settings in China. *JAMA Intern Med*. 2014;174(12):1914. doi:10.1001/jamainternmed.2014.5214

4. Lambert H, Shen X, Chai J, et al. Prevalence, drivers and surveillance of antibiotic resistance and antibiotic use in rural China: Interdisciplinary study. *PLOS Global Public Health*. 2023;3(8):e0001232. doi:10.1371/journal.pgph.0001232

5. O’Connor R, O’Doherty J, O’Regan A, Dunne C. Antibiotic use for acute respiratory tract infections (ARTI) in primary care; what factors affect prescribing and why is it important? A narrative review. *Irish Journal of Medical Science (1971 -)*. 2018;187(4):969-986. doi:10.1007/s11845-018-1774-5

6. Pratt MTG, Abdalla T, Richmond PC, et al. Prevalence of respiratory viruses in community-acquired pneumonia in children: a systematic review and meta-analysis. *Lancet Child Adolesc Health*. 2022;6(8):555-570. doi:10.1016/S2352-4642(22)00092-X

7. Jung C, Levy C, Béchet S, et al. Impact of C-reactive protein point-of-care testing on antibiotic prescriptions for children and adults with suspected respiratory tract infections in primary care: a French patient-level randomized controlled superiority trial. *Clinical Microbiology and Infection*. 2024;30(12):1553-1558. doi:10.1016/j.cmi.2024.07.014

8. Huang Y, Chen R, Wu T, Wei X, Guo A. Association between point-of-care CRP testing and antibiotic prescribing in respiratory tract infections: a systematic review and meta-analysis of primary care studies. *British Journal of General Practice*. 2013;63(616):e787-e794. doi:10.3399/bjgp13X674477

9. Do NTT, Vu TVD, Greer RC, et al. Implementation of point-of-care testing of C-reactive protein concentrations to improve antibiotic targeting in respiratory illness in Vietnamese primary care: a pragmatic cluster-randomised controlled trial. *Lancet Infect Dis*. 2023;23(9):1085-1094. doi:10.1016/S1473-3099(23)00125-1

10. Cals JWL, Butler CC, Hopstaken RM, Hood K, Dinant GJ. Effect of point of care testing for C reactive protein and training in communication skills on antibiotic use in lower respiratory tract infections: cluster randomised trial. *BMJ*. 2009;338(may05 1):b1374-b1374. doi:10.1136/bmj.b1374

11. Do NTT, Ta NTD, Tran NTH, et al. Point-of-care C-reactive protein testing to reduce inappropriate use of antibiotics for non-severe acute respiratory infections in Vietnamese primary health care: a randomised controlled trial. *Lancet Glob Health*. 2016;4(9):e633-e641. doi:10.1016/S2214-109X(16)30142-5

12. Althaus T, Greer RC, Swe MMM, et al. Effect of point-of-care C-reactive protein testing on antibiotic prescription in febrile patients attending primary care in Thailand and Myanmar: an open-label, randomised, controlled trial. *Lancet Glob Health*. 2019;7(1):e119-e131. doi:10.1016/S2214-109X(18)30444-3

13. Schot MJ, Van den Bruel A, Broekhuizen BD, et al. Point-of-care C-reactive protein to assist in primary care management of children with suspected non-serious lower respiratory tract infection: a randomised controlled trial. *BJGP Open*. 2018;2(3):bjgpopen18X101600. doi:10.3399/bjgpopen18X101600

14. Levinson T, Wasserman A. C-Reactive Protein Velocity (CRPv) as a New Biomarker for the Early Detection of Acute Infection/Inflammation. *Int J Mol Sci*. 2022;23(15):8100. doi:10.3390/ijms23158100

15. Smedemark SA, Aabenhus R, Llor C, Fournaise A, Olsen O, Jørgensen KJ. Biomarkers as point-of-care tests to guide prescription of antibiotics in people with acute respiratory infections in primary care. *Cochrane Database of Systematic Reviews*. 2022;2022(10). doi:10.1002/14651858.CD010130.pub3

16. Zou S, Liu J, Yang Z, Xiao D, Cao D. SAA and CRP are potential indicators in distinction and severity assessment for children with influenza. *International Journal of Infectious Diseases*. 2021;108:357-362. doi:10.1016/j.ijid.2021.05.057

17. Zhang Y, Zhang J, Sheng H, Li H, Wang R. Acute phase reactant serum amyloid A in inflammation and other diseases. In: ; 2019:25-80. doi:10.1016/bs.acc.2019.01.002

18. MALLE E, DE BEER FC. Human serum amyloid A (SAA) protein: a prominent acute‐phase reactant for clinical practice. *Eur J Clin Invest*. 1996;26(6):427-435. doi:10.1046/j.1365-2362.1996.159291.x

19. Perez L. Acute phase protein response to viral infection and vaccination. *Arch Biochem Biophys*. 2019;671:196-202. doi:10.1016/j.abb.2019.07.013

20. Chan AW, Tetzlaff JM, Altman DG, et al. SPIRIT 2013 Statement: Defining Standard Protocol Items for Clinical Trials. *Ann Intern Med*. 2013;158(3):200. doi:10.7326/0003-4819-158-3-201302050-00583

21. Cane J, O’Connor D, Michie S. Validation of the theoretical domains framework for use in behaviour change and implementation research. *Implementation Science*. 2012;7(1):37. doi:10.1186/1748-5908-7-37

22. French SD, Green SE, O’Connor DA, et al. Developing theory-informed behaviour change interventions to implement evidence into practice: a systematic approach using the Theoretical Domains Framework. *Implementation Science*. 2012;7(1):38. doi:10.1186/1748-5908-7-38

23. Wei X, Zhang Z, Walley JD, et al. Effect of a training and educational intervention for physicians and caregivers on antibiotic prescribing for upper respiratory tract infections in children at primary care facilities in rural China: a cluster-randomised controlled trial. *Lancet Glob Health*. 2017;5(12):e1258-e1267. doi:10.1016/S2214-109X(17)30383-2

24. Chinese Association of Integrated Traditional and Western Medicine: Laboratory Medicine Professional Committee. Expert Consensus on the Clinical Use of Serum Amyloid A in Infectious Diseases. *Chinese Journal of Laboratory Medicine*. 2019;42(3):186-192. doi:10.3760/cma.j.issn.1009-9158.2019.03.008

25. Hansson L, Hedner T, Dahlöf B. Prospective Randomized Open Blinded End-point (PROBE) Study. A novel design for intervention trials. *Blood Press*. 1992;1(2):113-119. doi:10.3109/08037059209077502

26. Richard J. Hayes, Lawrence H. Moulton. *Cluster Randomised Trials (2nd Ed.)*. Chapman and Hall/CRC; 2017. doi:10.4324/9781315370286

27. Pedroza C, Truong VT. Performance of models for estimating absolute risk difference in multicenter trials with binary outcome. *BMC Med Res Methodol*. 2016;16(1):113. doi:10.1186/s12874-016-0217-0

28. Tong A, Sainsbury P, Craig J. Consolidated criteria for reporting qualitative research (COREQ): a 32-item checklist for interviews and focus groups. *International Journal for Quality in Health Care*. 2007;19(6):349-357. doi:10.1093/intqhc/mzm042

29. McCambridge J, Witton J, Elbourne DR. Systematic review of the Hawthorne effect: New concepts are needed to study research participation effects. *J Clin Epidemiol*. 2014;67(3):267-277. doi:10.1016/j.jclinepi.2013.08.015
